# Using the Socio-Technical Allocation of Resources (STAR) approach to support chronic obstructive pulmonary disease management resource allocation in integrated care systems in England

**DOI:** 10.1007/s43999-025-00083-z

**Published:** 2025-12-16

**Authors:** Jack Ettinger, Sophie Hodges, Luca Ricci-Pacifici, Patrick J. O. Covernton, Wayne Smith, Andi Orlowski

**Affiliations:** Health Economics Unit, London, UK

**Keywords:** Chronic obstructive pulmonary disease (COPD), Resource allocation, Socio-Technical Allocation of Resources (STAR), Decision conferencing, Cost-effectiveness analysis, Healthcare pathway optimization, Value for money

## Abstract

**Background:**

Management pathways for chronic obstructive pulmonary disease (COPD) are complex, and stakeholders may need to consider different approaches that improve health and reduce inequalities, while also delivering value for money. We used the Socio-Technical Allocation of Resources (STAR) approach to identify opportunities for improving COPD resource allocation in five integrated care systems (ICS) in England.

**Methods:**

STAR uses decision conferencing involving facilitated workshops and a decision analysis model of participants’ perspectives. Two facilitated workshops involving key COPD management stakeholders were undertaken in each ICS. This allowed participants to gain an understanding of the value of the current care pathway and use this as a discussion point alongside an understanding of the ICSs priorities and patient preference to define a shortlist of potential pathway improvements. Modelling was then undertaken to understand the expected net costs and net health benefits of each pathway improvement, and these were then ranked.

**Results:**

Potential COPD pathway improvements were identified within each ICS. These differed across the five ICSs based on local population and ICS priorities, but included: (1) more effective use of the virtual ward; (2) promoting additional respiratory services through social prescribing; (3) proactive case-finding/increased screening; (4) very brief advice for tobacco dependency; (5) increasing uptake of pulmonary rehabilitation services; (6) introducing patient COPD apps; (7) increasing uptake of smoking cessation services; and (8) conducting patients’ yearly reviews through group consultations. Implementing the top-ranked interventions was predicted to provide notable improvements in COPD population health benefit while having a cost saving, neutral or minimal budget impact.

**Conclusion:**

The STAR approach could provide a valuable resource allocation decision tool at a local level and consider potential areas for improving health outcomes while minimising budget impact. Such findings could be used to support decisions on where best to allocate resources in ICS disease programmes.

**Supplementary Information:**

The online version contains supplementary material available at 10.1007/s43999-025-00083-z.

## Key messages

What is already known on this topic

• Chronic obstructive pulmonary disease (COPD) imparts a significant resource burden on the NHS in England and chronic respiratory disease is one of the key clinical areas of health inequality that require accelerated improvement, making this a principal goal at the Integrated Care System (ICS) level.

• ICSs need decision-making methods that can allow them to systematically and transparently make decisions aimed at improving population health and value for money in a structured way.

What this study adds

• This study shows that the Socio-Technical Allocation of Resources (STAR) approach can provide a framework for optimising resource allocation for the COPD management pathways in ICSs in England.

• The approach shows that there are opportunities for improving COPD population health benefit with minimal budget impact or even cost savings.

How this study might affect research, practice or policy

• This work provides a methodology that ICSs can use in their decisions on where best to allocate resources in ICS respiratory programmes. The approach could be extended beyond the ICSs involved in this study and also to other therapy areas.

**Supplementary Information:**

The online version contains supplementary material available at 10.1007/s43999-025-00083-z.

## Introduction

Chronic obstructive pulmonary disease (COPD) is a heterogeneous lung condition associated with abnormalities of the airways (bronchitis, bronchiolitis) and/or alveoli (emphysema) that cause persistent, often progressive, airflow obstruction, resulting in chronic respiratory symptoms (dyspnoea, cough, expectoration, exacerbations) [1]. These symptoms have a substantial negative impact on patients’ physical activity, health status and quality of life [1–3]. Furthermore, COPD is the third leading cause of death worldwide (excluding COVID-19) behind ischaemic heart disease and stroke, and the sixth leading contributor to disease burden in terms of disability-adjusted life years (GBD 2021 Diseases and Injuries Collaborators, 2024) [4]. 

Approximately 1.4 million people in England have a diagnosis of COPD and approximately 500,000 are potentially undiagnosed [5]. Consequently, COPD imparts a significant resource burden on the NHS in England. For instance, there were approximately 4.25 million hospital admissions for COPD in England and Wales between 1999 and 2020 [6], and direct costs due to COPD in England are expected to exceed £2.3 billion per year by 2030 [7]. 

Management of COPD is complex, requiring a multidisciplinary approach, signposting, psychological support, community activation, self-management and comorbidity considerations [8, 9]. Furthermore, there are a number of modifiable risk factors (especially smoking) for COPD and common comorbid conditions that provide opportunities for primary prevention and long-term cost-savings [9–11]. Key components of the COPD management pathway include ensuring early detection with accurate diagnosis and optimizing long-term management to reduce exacerbations, hospital admissions and premature mortality, and pulmonary rehabilitation [8]. Unfortunately, interventions with clear evidence of benefit may be underutilised and care models may be suboptimal (Agusti et al., 2023b) [12]. For example, despite overwhelming evidence to support its use, only a small proportion of patients in England undergo early pulmonary rehabilitation following hospitalisation due to low rates of referral and poor uptake by patients [12–14]. 

Recent organisational reforms in the NHS in England led to the formation of 42 integrated care systems (ICSs). These are partnerships that bring together NHS organisations, local authorities and others to take collective responsibility for planning services, improving health and reducing inequalities, while also attempting to deliver value for money, across defined geographical areas [15, 16]. The NHS faces the challenge of limited growth in its budgets meaning ICSs need to reallocate existing resources to improve the efficiency of their services. ICSs need decision-making methods that can allow them to systematically and transparently make decisions aimed at improving population health and value for money in a structured way [16, 17]. In particular, the Core20PLUS5 initiative from NHS England identified chronic respiratory disease as one of the five key clinical areas of health inequality that require accelerated improvement at a national and system level, making this a principal goal at the ICS level [18]. 

**Fig. 1:**
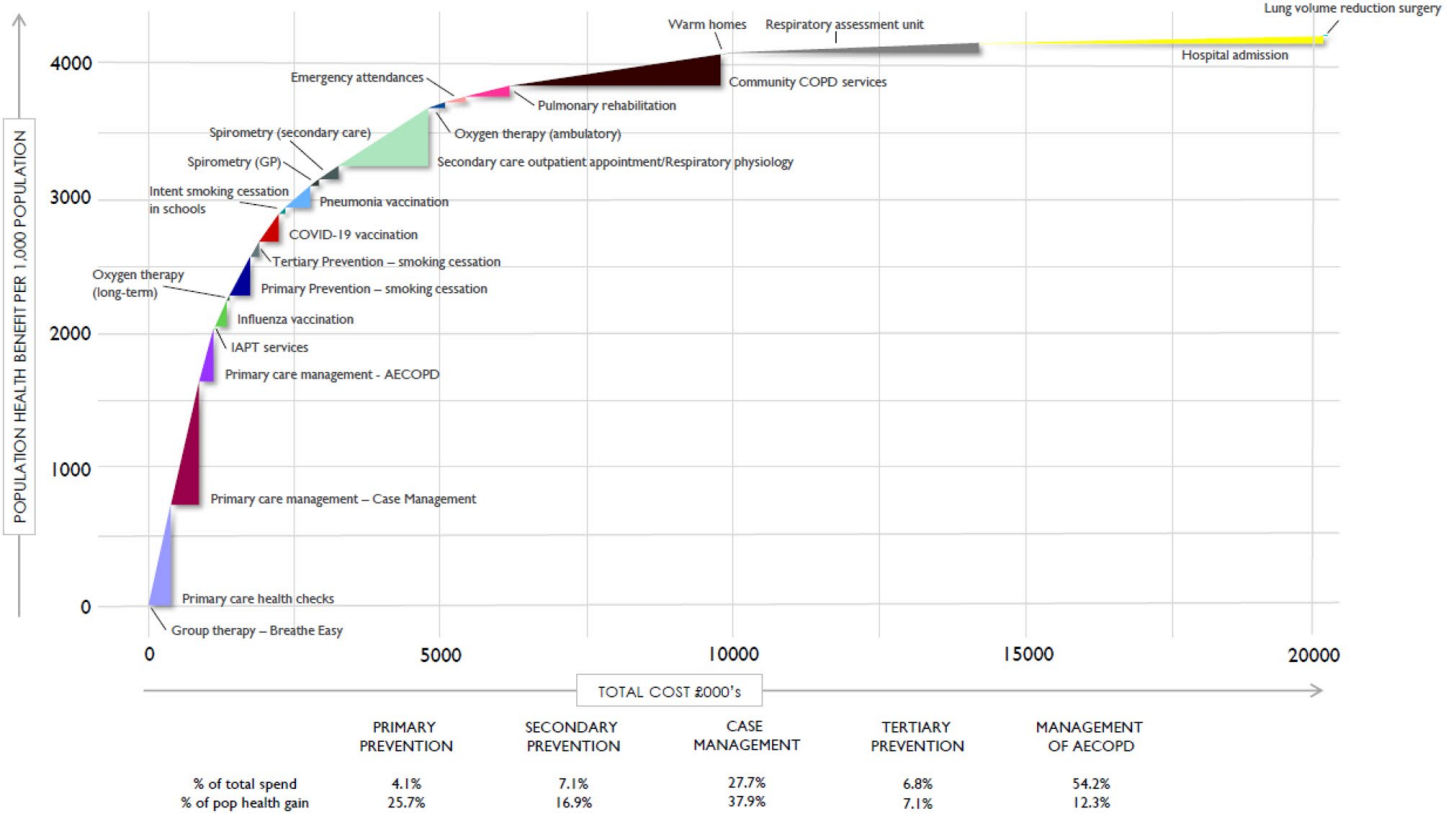
Population health benefit versus costs in Nottingham and Nottinghamshire ICS. Abbreviations: AECOPD, acute exacerbation of COPD; COPD, chronic obstructive pulmonary disease; GP, general practice; IAPT, Improving Access to Psychological Therapies; ICS, Integrated Care System

Socio Technical Allocation of Resources (STAR) is a form of deliberative cost-effectiveness analysis that emphasises visual models (Fig. 1 and supplementary Fig. 4a-d) [19]. Building upon other decision analysis techniques such as programme budgeting and marginal analysis (PBMA) and multiple criteria decision making (MCDA), STAR provides a pragmatic, structured way to bring stakeholders together to think about allocating resources across the entirety of a disease pathway [19, 20]. It allows groups to systematically identify and rank their preferences using criteria including population health benefit, potential to reduce health inequalities, and cost to the system clinician or patient [21]. The STAR approach has been used for cost-effectiveness analyses to prioritise interventions within public health programmes, including the NHS [19, 22, 23]. The STAR approach supports commissioners in identifying budget prioritisations and making resource allocation decisions in a collaborative manner [24]. 

In the current study, we used the STAR approach to look at resource allocation in the COPD management pathways in five ICSs in England. To the best of the authors knowledge, this is the first time the STAR approach has been used since the introduction of ICSs in 2022. STAR was chosen over other decision analysis tools such as PBMA and MCDA because of its focus on collaboration in a local context, a key challenge for ICSs [16, 17, 19, 20]. The aim of this study was two fold. Firstly, to assess the relative value for money of the different interventions in the COPD pathway in each ICS, thereby identifying key opportunities for improving COPD prevention and create a priority list of pathway improvements that could be implemented in each ICS based on value for money. Secondly, we aimed to trial a method for resource allocation that could be applied more widely across ICSs and disease areas.

## Methods

**Fig. 2:**
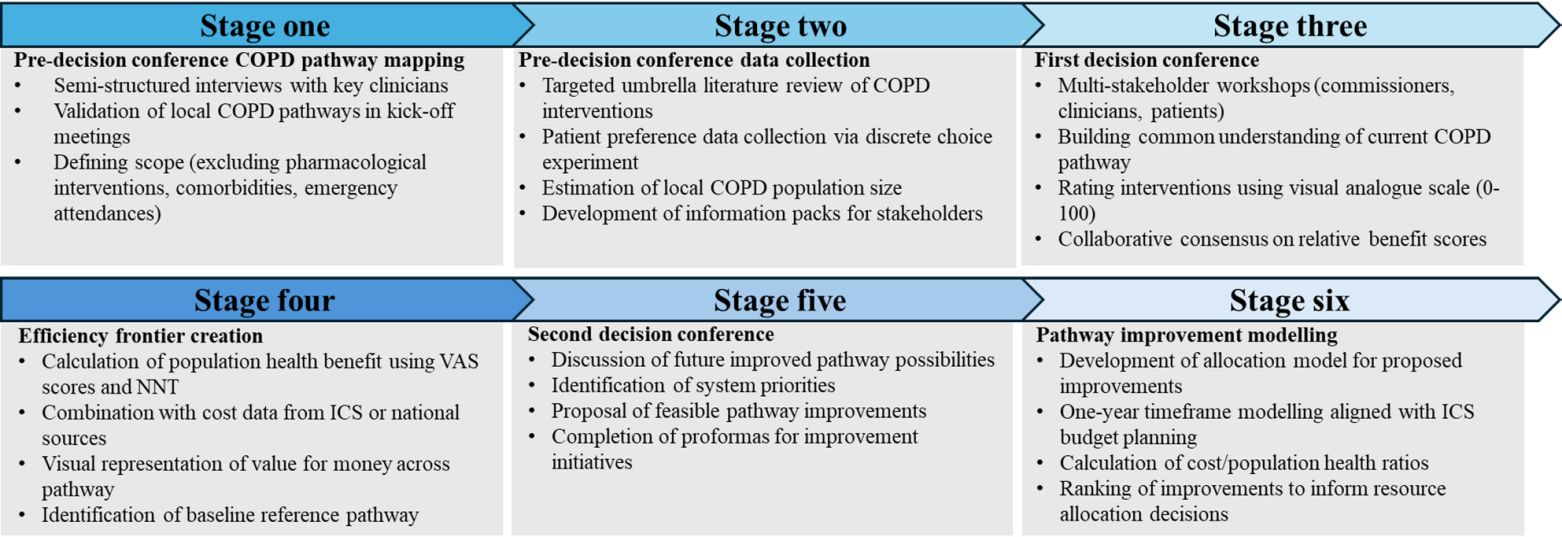
STAR Implementation process. ICS, integrated care system; COPD, chronic obstructive pulmonary disease; VAS, visual analogue scale; NNT, number needed to treat; PHB, population health benefit; STAR, Socio-Technical Allocation of Resources

The Socio-Technical Allocation of Resources (STAR) approach was implemented through a structured process involving multiple stakeholders across five integrated care systems (ICSs) in England (Fig. 2). This approach uses decision conferencing to help key stakeholders align on important issues within their organisation through facilitated workshops and decision analysis modelling [25]. 

### Study population

An invitation was sent out to respiratory programme teams in the Midlands to join the programme. The Midlands region was chosen as the area covered by the funding body. The respiratory programmes that volunteered to this pilot study were : Birmingham and Solihull, Coventry Place (part of Coventry and Warwickshire ICS), Gloucestershire, Northamptonshire, and Nottingham and Nottinghamshire. They represented a ‘convenience sample’ of ICSs as participation was voluntary.

### Stage one: Pre-decision conference COPD pathway mapping

Semi-structured interviews were undertaken with key clinicians involved in each ICS’s respiratory programme, aiming to build an understanding of the local COPD pathway. These pathways were summarised and validated in kick-off meetings held with each ICS (supplemental Fig. 1). Overtreatment and inappropriate prescribing have been highlighted in the literature as large issues driving costs and poor outcomes in people with COPD [26, 27]. That said, pharmacological interventions for the stable management of COPD were not part of the decision problem at hand. ICSs already had in place medicine optimisation programmes looking at prescribing practices. In addition, it is not possible for the ICS respiratory programmes to determine individual prescribing practises and funding decisions which are made by NICE [28]. Treatment of comorbidities and emergency attendances (not leading to hospital admission) were also out of scope.

### Stage two: Pre-decision conference data collection

Two data collection activities were undertaken prior to the decision conferences to gather data that would be required for the STAR model and to build and understanding of patient preferences for COPD care.

A targeted umbrella review (exploring previously published systematic literature reviews and network meta-analyses) was carried out in July-Aug 2022 to investigate the effects of a broad range of clinical, surgical, behavioural, environmental, and socioeconomic interventions for COPD symptoms and/or progression. This information was used to support participants in their valuation of the health benefits of each intervention, the number who benefit for each intervention and the expected resource use reduction from pathway improvements. The health benefits of each identified intervention were determined in terms of the reported outcomes, and this was used to estimate the relative benefit and numbers needed to treat (NNT) for use in the STAR model. A simplifying assumption is made in the STAR model that the benefit of each intervention is independent from the other interventions in the pathway. However, participants in the decision conferences were encouraged to discuss interdependencies when valuing the interventions.

Information on patient preferences for accessing care was based on a discrete choice experiment performed in partnership with IPSOS, a research organisation, in 520 patients with a diagnosis of COPD across the five ICSs; the surveys were completed in Aug-Sep 2022 [29]. Preference was also informed by patients, carers and representatives of patient groups participating in the decision conferences. This allowed all participants to understand the impact of changing or adding to the pathway on preferences in both a quantitative (the discrete choice experiment) and qualitative (through patient input). Estimates for the size of the COPD population in each ICS were based on government health statistics and published literature (supplemental Fig. 2).

The collected evidence was incorporated into an information pack to prepare stakeholders for the decision conferences, ensuring participants had an understanding of the STAR process and the local COPD context. The information pack included data on the local population, preferences of patients with stable COPD, evidence from the umbrella literature review on quality-adjusted life years (QALYs) of interventions in the current care pathway and interventions that impact the wider determinants of health.

### Stage three: First decision conference

The first in-person half-day collaborative decision conferences were undertaken in each ICS from 11th October 2022 to 20th December 2022. Each decision conference was attended by local stakeholders nominated by each ICS’s respiratory programme, and included commissioners (responsible for allocating budget), finance representatives, clinicians involved in the ICS respiratory programmes specialising in respiratory medicine (covering primary, community and secondary care), local authority representatives, public health professionals, and patient representatives (either patients with COPD or volunteers from the charity Breathing Space). Approximately 20 individuals attended each decision conference. ICSs were given guidance on who they should include in the decision conferences but had the freedom to choose their own stakeholders. They were supported in finding patient representatives if they were unable to find them directly.

The first decision conference focussed on building a common understanding among participants of the current COPD pathway, including the population at risk of developing COPD or already living with COPD, and the relative value of all the COPD pathway interventions.

To inform the STAR model, participants were asked to rate (on a 0–100 visual analogue scale [VAS]) the relative benefit (value), in terms of length and quality of life, for each intervention and programme in the pathway (supplemental Fig. 3) assuming successful implementation, to the typical beneficiary, compared with current care [19, 30]. Based on the information pack, their local experience and clinical expertise, participants were asked to identify the option that would deliver the greatest individual health benefit, in terms of length and quality of life, and this was assigned a score of 100. They then scored each remaining intervention relative to this benchmark score of 100 and a fixed benchmark of 0 (corresponding to no additional health benefit) compared with current care. A single final score for each option was decided upon through facilitated collaborative discussions within the workshop. The facilitators encouraged the group to take the advise of the patient representatives and the respiratory clinicians in the decision conference in the case of disagreement. This method was specifically chosen to allow variation between ICSs to reflect their different populations and the local interventions.

### Stage four: Efficiency frontier creation

The output of the first decision conference, the relative benefit score derived from the VAS ratings, was combined with information on activity provided by the ICS respiratory programme’s databases (the number treated) and the NNT identified through the umbrella literature review to calculate the population health benefit. This was then combined with costs either provided by the ICS or national data to build a STAR “efficiency frontier”, a visual representation of the value for money of the COPD pathway (Fig. 1 and supplementary Fig. 4A-D). The calculations for the efficiency frontier are explained in the supplemental appendix. This provided the baseline reference pathway for consideration of the impact of pathway improvement modelling.

Any data required for the efficiency frontier that could not be provided in the decision conference itself was sought between the first and second conference.

#### Interpreting the STAR efficiency frontier

Each intervention was represented visually by a triangle with costs on the x-axis and population health benefit on the y-axis (see supplemental appendix for background information on how to interpret the efficiency frontier). Tall and narrow triangles represent relatively more cost-effective interventions than short and wide triangles. The triangles were ordered in a sequence according to their cost-effectiveness (from high to low) to display the efficiency frontier (see Fig. 1 for an example, with efficiency frontiers from all of the ICSs in supplementary Fig. 4). The purpose of the efficiency frontier was to help participants think about how the care pathway for COPD could be developed. The aim was to move the efficiency frontier to the left and upwards, thus reducing costs and improving the population health benefit of the pathway, and optimising how healthcare resources can best be allocated across the system to maximise outcomes for the local population.

### Stage five: Second decision conference

The second decision conferences, held between the 1st January and 9th March 2023, focussed on what the future improved pathway might look like and highlighting the main challenges. Following discussion on the priorities for the system, participants were asked to propose pathway improvements that could feasibly be implemented — this was done by completing proformas in the information pack. The improvements could consist of multiple interventions and could be new interventions or changes to how a current intervention is implemented. Participants were asked to describe the following: (1) the population segment that would receive the intervention for each initiative; (2) of those that receive the intervention, who would likely benefit; (3) the health benefits that those who do benefit would likely receive; and (4) the relative value of the intervention compared to those modelled in the current care pathway (if required).

### Stage six: Pathway improvement modelling

Following the decision conferences, an allocation model was developed to prioritise the proposed pathway improvements adapted from the methodology proposed by Airoldi et al. [19] For each ICS, the model calculated the health benefits and costs for each pathway improvements, allowing direct comparison.

The timeframe in which the expected changes would be realised differed depending on the particular pathway improvement. To align to the budget planning of ICSs, the expected change that could occur over a period of one year was modelled for each pathway improvement. Different scenarios were included where there were multiple possibilities for implementing the pathway improvement or where there was uncertainty around how the improvement could be implemented. For example, increasing uptake of smoking cessation services could be implemented by either increasing capacity or standardising referral pathways and could have differing levels of uptake among the target population.

For each pathway improvement, the number who would benefit in a given year was calculated using the NNT for the intervention, identified from the umbrella literature review, and the number of people expected to be treated in a given year. Then, we calculated the additional relative population health benefit by multiplying the number of people who would benefit by the expected improvement in their health (based on the relative benefit scores from the decision conferences). We then calculated the additional costs of implementing the intervention and subtracted any expected cost savings from reduced healthcare resource use elsewhere in the system (such as fewer hospital admissions). The formulae used to build the allocation model are further explained in the supplementary appendix.

Modelled improvements were ranked based on the cost/population health ratio and recommended improvements for the COPD pathway were based on this ranking. This information was provided to the ICS respiratory programmes to inform their resource allocation decisions.

## Results

In all ICSs, primary prevention of COPD through smoking cessation was identified as the option that provided the greatest individual benefit and represented the benchmark maximum benefit score of 100 (supplemental Fig. 3; supplemental Tables 1–5). Efficiency frontiers constructed for each ICS varied based on their local population and system priorities (Fig. 1; supplemental Fig. 4; supplemental Tables 1–5). Across the 5 ICS, hospital admissions were generally the greatest contributor to total costs and were among the least cost-effective interventions, the exception being Birmingham Solihull, where they were exceeded by hospice-provided palliative care. At the other end of the scale, primary care case management and primary care management of exacerbations were among the most cost-effective interventions.

### Modelled pathway improvements

Based on the consensus reached in second decision conference, interventions that might improve the pathway were identified, modelled and ranked for their impact on cost/population health ratio (Tables 1, 2, 3, 4 and 5). For example, improving case finding by targeted COPD screening in Nottingham and Nottinghamshire ICS was estimated to be both health-generating and cost-saving in both the most optimistic and least optimistic scenarios (Fig. 1B). In the most optimistic scenario, the cost ratio was 3.80, meaning that it was predicted to save £3.80 for every £1.00 spent elsewhere in the COPD pathway. The additional cost/additional population health ratio was 5.28, meaning that it was predicted to save £5.28 for every additional unit of population health benefit it generates.

**Table 1:** Modelled pathway improvements ranked by cost/population health ratio: Birmingham and solihull ICS

Rank	Pathway improvement (scenario)	PHB gain	Total additional pathway costs (£)	Cost ratio	Cost/population health ratio
1	*Expanding palliative care through hospice-at-home virtual ward (compared to hospice care)* ^*a*^	*10*,*675*	*-11*,*312*,*029*	*27.86*	*-1059.67*
2	Virtual ward as early discharge support	48,930	-1,802,830	2.08	-36.85
3	Increasing uptake and quality of spirometry testing (doubling tests in primary care)	30,675	-134,401	1.89	-4.38
4	Increasing uptake and quality of spirometry testing (expanding RDHs)	43,575	-163,743	2.23	-3.76
5	Promoting self-care through the myCOPD app^b^	450,680	5,868	N/A	0.01
6	Increasing uptake of smoking cessation services (tertiary prevention—Quit with Bella app)	143,370	33,474	0.71	0.23
7	Promoting respiratory services within localities through social prescribing—physical activity	116,220 290,615 581,360	30,016 (10%) 77,551 (25%) 155,143 (50%)	0.68	0.26
8	Increasing uptake of smoking cessation services (primary prevention—Quit with Bella app)	13,900	7,508	0.25	0.54
9	Improving the quality of primary care management through clinical education	139,640	167,064	N/A	1.20
10	Driving pneumonia vaccination uptake	188,325	489,078	0.19	2.60
11	Increasing uptake of PR services (increasing completion rate to 75%)	33,286	145,254	0.16	4.36
12	Increasing uptake of PR services (double people starting the course)	22,606	155,218	0.11	6.87
13	Psychological support	88,050 220,050 440,100	766,497 (10%) 1,919,475 (25%) 3,836,326 (50%)	0.08	8.71
14	*Expanding palliative care through hospice-at-home virtual ward (compared to GP palliative care)*	*10*,*675*	*133*,*944*	*0.68*	*12.55*
15	Increasing uptake of smoking cessation services (primary prevention—smoking cessation service)	390,100	5,561,830	0.01	14.26
16	Increasing uptake of smoking cessation services (tertiary prevention—smoking cessation service)	210,690	3,250,792	0.04	15.43
17	Virtual ward admissions avoidance	11,445 28,595 57,190	504,054 (10%) 1,217,036 (25%) 24,405,820 (50%)	N/A	42.07 42.56 44.04

Recommended pathway improvements are highlighted

^a^This improvement has not been explicitly recommended, as there is uncertainty over how it would be implemented and what the relevant comparator (GP, community or hospice-provided care) would be, all of which would impact the cost/population health ratio

^b^myCOPD is an app that supports patients to self-manage their condition and receive information from their care providers

COPD, chronic obstructive pulmonary disease; N/A, not available; PHB, population health benefit; PR, pulmonary rehabilitation; RDH, respiratory diagnostic hub

**Table 2:** Modelled pathway improvements ranked by cost/population health ratio: Coventry place

Rank	Pathway improvement (scenario)	PHB gain	Total additional pathway costs (£)	Cost ratio	Cost/population health ratio
1	Expansion of the virtual ward	24,820	-553,523	2.72	-22.30
2	Targeting spirometry testing and improving uptake (improving the diagnosis rate to 56.28%)	39,690	-171,982	3.44	-4.33
3	Targeting spirometry testing and improving uptake (improving the diagnosis rate to 35%)	24,696	-80,714	2.15	-3.27
4	Targeting spirometry testing and improving uptake (increasing capacity to meet demand)	19,894	-52,051	1.74	-2.62
5	Targeting spirometry testing and improving uptake (expanding TLHCs)	10,192	-26,258	1.72	-2.58
6	Joint clinics in primary care (with current establishment of 5.6 RNSs)	174,604	37,714	0.56	0.22
7	Education package for people with COPD	65,520	33,251	N/A	0.51
8	Joint clinics in primary care (with 8.6 RNSs)	447,128	381,796	-1.05	0.85
9	Education in schools against smoking and vaping	97,700	107,939	0.41	1.10
10	Carer support	4,800	10,080	N/A	2.10
11	Targeted awareness campaign	24,946	143,629	0.46	5.76
12	Innovation in smoking cessation services (increasing capacity in Healthy Lifestyles service)	140,032	1,372,001	0.08	9.80
13	Innovation in smoking cessation services (improving quit rates in GP-and pharmacy-led services	10,192	189,478	0.04	18.59

Recommended pathway improvements are highlighted

COPD, chronic obstructive pulmonary disease; PHB, population health benefit; RNS, respiratory nurse specialist; TLHC, targeted lung health check

The proposed interventions and their impact differed by ICS due to variations in local population and system priorities, although there were some common features. For example, in three of the ICSs, improvements involving the use of a virtual ward were both health-generating and cost-saving (Tables 1, 2 and 3).

**Table 3:** Modelled pathway improvements ranked by cost/population health ratio: Gloucestershire ICS

Rank	Pathway improvement (scenario)	PHB gain	Total additional pathway costs (£)	Cost ratio	Cost/population health ratio
1	More effective use of the virtual ward	20,212	-385,821	1.91	-19.09
2	Proactive case finding (most optimistic scenario)	93,075	-639,803	7.30	-6.87
3	Increasing uptake of PR (online offering)	18,000	-11,874	4.87	-0.66
4	Improving uptake to Mindsong^a^ and KiActiv^b^	6,390	-2,491	N/A	-0.39
5	VBA for tobacco dependency	12,960	-399	1.06	-0.03
6	Vaping as a harm reduction pilot	41,515 743,850	13,687 (non-quitters) 245,532 (10%)	0.15	0.32 0.33
7	Improving uptake of pneumonia vaccinations	34,200	16,082	0.56	0.47
8	MPT management of patients (1 PCN)	54,320	101,559	N/A	1.87
9	MPT management of patients (2 PCNs)	90,440	203,118	N/A	2.25
10	Proactive case finding (least optimistic scenario)	5,475	12,919	0.77	2.36
11	MPT management of patients (3 PCNs)	116,830	304,677	N/A	2.61
12	Increasing uptake of smoking cessation services	107,800	323,060	0.02	3.00
13	Avoiding fuel poverty	1,350 3,420 6,750	9,000 (10%↑) 22,800 (20%↑) 45,000 (50%↑)	N/A	6.67
14	Psychological support for patients in primary care	46,050 115,200 230,400	366,864 (10%) 917,845 (25%) 1,835,823 (50%)	0.03	7.97
15	Increasing uptake of PR (improving completion rates in the current services)	23,490	266,273	0.07	11.34
16	Increasing uptake of PR (improving uptake through the standard route)	18,000	271,255	-0.05	15.07

Recommended pathway improvements are highlighted

^a^Mindsong is a ‘breath in, sing out course’ for people with chronic lung conditions

^b^KiActiv is a personalised online physical activity course for patients who are referred by primary, community and secondary care

MPT, multi-professional team; PCN, primary care network; PHB, population health benefit; PR, pulmonary rehabilitation; VBA, very brief advice

### Recommended pathway improvements

Based on the cost/population health ratio ranking, several pathway improvements were identified that were likely to lead to the greatest additional population health benefit per pound spent within each ICS (Tables 1, 2, 3, 4 and 5). These differed across the five systems according to local factors, but among the recommendations were: (1) more effective use of the virtual ward; (2) promoting additional respiratory services through social prescribing; (3) proactive case finding/increased screening; (4) very brief advice for tobacco dependency; (5) increasing uptake of pulmonary rehabilitation services; (6) introducing the myCOPD app; (7) increasing uptake of smoking cessation services; and (8) conducting patients’ yearly reviews through group consultations. Virtual interventions such as the virtual ward and myCOPD app tended to rank highly due to their low cost per person, even though the number who benefit was small.

In the Birmingham and Solihull ICS, implementing all recommendations could save around £1.9-2.0 million per year alongside an increase in total population health. In Coventry Place, the recommended expansion of the virtual ward was estimated to save £0.55 million per year, which would be sufficient to cover most of the additional cost of other improvements (£0.56 million for those ranked 6–9) (Table 2). In the Gloucestershire ICS, implementing all the recommendations was estimated to save £1.04 million per year along with an increase in total population health (Table 3). In the Northamptonshire ICS, post-exacerbation support would be expected to save approximately £47,000 per year due to the reduction in readmissions; the other recommendations were either cost neutral or associated with only minimal costs but would be expected to provide population health benefits (Table 4). The top-ranked recommendation in Nottingham and Nottinghamshire ICS involved use a successful targeted screening programme (see above) (Table 5).

**Table 4:** Modelled pathway improvements ranked by cost/population health ratio: Northamptonshire ICS

Rank	Pathway improvement (scenario)	PHB gain	Total additional pathway costs (£)	Cost ratio	Cost/population health ratio
1	Post-exacerbation support	12,880	-46,757	1.15	-0.28
2	Reducing unwarranted variation in primary care yearly reviews through group consultations (all scenarios)	79,200 198,780 397,440	-179 (10%) -14 (25%) -29 (50%)	1.00 1.00 1.00	0.00 0.00 0.00
3	Launch of myCOPD app^a^	367,850	3,832	∞	0.01
4	VBA in primary care	6,525	1,956	0.70	0.29
5	Improved signposting to services through information centres	100,904	47,433	0.41	0.47
6	Increasing capacity in spirometry testing	60,760	33,973	0.91	0.56
7	Increasing uptake of smoking cessation services (increasing capacity)	83,525	254,493	0.15	3.05
8	Increasing uptake of smoking cessation services (standardised referral pathways)	21,450	85,964	0.14	4.01
9	Expansion of PR services	1,932	25,836	0.08	13.37

Recommended pathway improvements are highlighted

^a^myCOPD is an app that supports patients to self-manage their condition and receive information from their care providers

COPD, chronic obstructive pulmonary disease; PHB, population health benefit; PR, pulmonary rehabilitation; VBA, very brief advice

**Table 5:** Modelled pathway improvements ranked by cost/population health ratio: Nottingham and Nottinghamshire ICS

Rank	Pathway improvement (scenario)	PHB gain	Total additional pathway costs (£)	Cost ratio	Cost/population health ratio
1	Improving case-finding by targeted COPD screening (most optimistic scenario)	254,505	-1,344,055	3.80	-5.28
2	Improving case-finding by targeted COPD screening (least optimistic scenario)	56,430	-196,521	1.94	-3.48
3	Making every contact count	23,814	-2,489	1.20	-0.10
4	Conducting patients’ yearly reviews through group consultations	206,040 516,290 1,032,580	32 (10%) 30 (25%) 61 (50%)	1.00	0.00
5	Introducing a referral pathway to Breathe Easy groups^a^	1,116 2,728 5,456	559 (10%) 1,365 (25%) 2,731 (50%)	N/A	0.50
6	Improving uptake to smoking cessation services (increasing uptake to meet the 5% target set by NICE)	102,628	69,018	0.47	0.67
7	Improving uptake to smoking cessation services (doubling the number of people with COPD who set quit dates)	106,624	76,403	0.45	0.72
8	Expanding the INTENT smoking prevention programme in schools	140,200	203,758	0.29	1.45
9	Offering a post-PR exercise course	8,100 20,250 40,490	19,800 (10%) 49,500 (25%) 99,220 (50%)	N/A	2.44
10	Expanding access to pulmonary rehabilitation	366,480 91,440	3,222,846 (MRC 3+) 806,954 (MRC 2+)	0.09	8.79
11	Expanding affordable warmth schemes	2,535 7,605	92,468 (×2) 277,403 (×4)	N/A	36.48

Recommended pathway improvements are highlighted

^a^Breathe Easy groups provide support and information for people living with a lung condition and for those who look after them

COPD, chronic obstructive pulmonary disease; MRC, Medical Research Council (Score); NICE, National Institute for health and Care Excellence; PHB, population health benefit; PR, pulmonary rehabilitation

## Discussion

The use of the STAR approach in the current analysis suggests that there are opportunities to optimise resource allocation in the COPD management pathway at a local level within individual ICSs. Implementing these opportunities was predicted to result in notable improvements in COPD population health benefit while having a cost saving, neutral or minimal budget impact. The modelled pathway improvements were developed to support decisions on where best to allocate resources by looking at how each pathway improvement could affect the allocation of resources across the entire COPD pathway. They are not meant to represent an accurate reflection of the costs and benefits of the COPD pathway pre- and post-improvement, nor do they represent a full economic evaluation of each pathway improvement. Instead, they provide a relative view of which interventions are most likely to be cost-effective, supporting decision making in the ICS. One ICS involved in the current study incorporated the findings into their budget planning for the subsequent financial year, thus demonstrating the practical value of this approach as a tool for supporting local decision making. This is important as ICSs have limited budgets and resources to support decision making, therefore pragmatic methods that bring stakeholders together, making best use of local evidence is of value.

The notable strength of this study was that ICS were able to assess the value of regional, small scale interventions alongside national priorities for their ICS respiratory programme based on cost-effectiveness. For example, areas with local organisations and initiatives were able to include them in the decision making framework. These included Mindsong (a breathing and singing course) and KiActiv (an online physical activity course) in Gloucestershire (Table 3), and the myCOPDapp in Northamptonshire ICS (Table 4) and Birmingham and Solihull ICS (Table 1). These interventions were then assessed against the virtual ward, a national initiative for COPD exacerbation management.

There are some weaknesses in the current analysis that should be acknowledged

Firstly, we used one method of valuing individual health benefit in the current analysis that was based on the methods employed by Airoldi et al. [19]. Other iterations of STAR use QALYs published in the literature [31, 32]. This method may give a more accurate picture of the absolute health gain generated from the interventions. The use of the relative benefit score, rather than a measure of absolute benefit such as the QALY means that, while the method is suitable for decision making in each ICS, the results are not comparable across ICSs. One benefit of the Airoldi et al. method is it allows stakeholders to debate the comparative benefit of each of the interventions [19]. This discussion in itself led to useful knowledge sharing among key stakeholders within the ICS, especially when patients were present, that would not have occurred if QALYs from the literature were used. There was also some consistency in the ratings between ICSs (supplementary Figs. 3 A-E). However, a key limitation with this method is that the composition of stakeholders participating in the decision conferences inevitably influences the valuation of each intervention. While we aimed for broad representation including commissioners, clinicians, finance representatives, and patient representatives, the specific mix of participants and their relative influence in discussions may have affected the valuation of interventions and the identification of pathway improvements.

Secondly, a simplifying assumption is made in the STAR model that the benefit of each intervention is independent from the other interventions in the pathway. However, participants in the decision conferences were encouraged to discuss interdependencies when valuing the interventions. In reality, the effectiveness of an intervention could depend on the other interventions provided, which could affect the overall population health benefit and cost-effectiveness ratios.

Thirdly, data was limited for certain interventions. QALYs were not readily available in the literature for prevention interventions and local programmes (such as the volunteer-led interventions). This meant participants had differing levels of prior information on the benefits of each intervention from the literature. There was also a lack of available data in the literature regarding the healthcare resource use of the pathway improvements. In most cases, the literature review only identified impacts on urgent care (hospitalisations and exacerbations) and impacts of improvements on other elements of the pathway are not known. Similarly, it was not possible to evidence the potential capital or programme costs that may be involved in the development of the pathway improvements within the timeframe of this project. Healthcare resource use would also have been influenced by COVID-19, which may alter finding compared with previous (or subsequent) years. These factors may have affected the cost/population health ratios if they were included.

Fourthly, as the COPD management pathway is complex with many components and potential areas for improvement, the decision conferences were intensive requiring a high degree of data collection, analysis and facilitation of the decision conferences. This limits the ability of ICS programme teams with limited available resource to be able to undertake the STAR process themselves.

Fifthly, as the NHS has a tariff-based payment system, it is not necessarily true that, at least in the short-run, resource would be released from reductions in hospital admissions. However, in a system with long waiting lists, it could mean that a bed can be used for another purpose other than a hospital admission for a COPD patient. A sustained reduction in activity, in the long run, would support the reallocation of resources away from the acute sector.

Finally, the exact value of primary prevention in the reduction of COPD cases is not known. We used the expected cost within a calendar year, as one year was the relevant timeframe for budget planning; however, stopping someone from developing COPD would have benefits beyond the one-year timeframe. According to a study in the US, the average life expectancy for someone from the time of diagnosis with COPD is 17.2 years [33]. Factoring this into the estimates would have increased the ranking of smoking cessation interventions.

The current analysis was performed in five selected ICSs in the Midlands of England yet has two broader points that are relevant for wider policy. Firstly, while the pathways and potential improvements would differ in other ICSs due to local population and system priorities, the principles of the STAR analysis in terms of identifying opportunities for more efficient resource allocation would apply equally to other parts of England and the UK. Secondly, some specific findings were common between ICSs in the current analysis, for example, the clear benefit of smoking cessation programmes to respiratory budgets and we might expect these to extend to other regions. The STAR approach would also be equally applicable to other disease pathways beyond COPD.

### Conclusions and implications for practice

The results of this analysis suggest that the STAR methodology can provide ICSs with a structured framework to identify potential opportunities for improving the value of respiratory and other services by giving insights into where to focus their efforts in an environment where the NHS is unlikely to see increased funding in real terms for the foreseeable future. The approach demonstrated how engaging a range of stakeholders in the decision-making process may result in less resistance to change and facilitate making difficult choices, such as decommissioning services. This mirrors the conclusions of similar approaches to optimising management pathways in eating disorders and back pain [32, 34]. While our study demonstrates the potential of the STAR approach as a decision-making tool, further research would be valuable to assess the long-term impact of implementing the identified pathway improvements on health outcomes and resource utilization.

## Supplementary Information

Below is the link to the electronic supplementary material.

Supplementary Material 1
