# Supplementary material for: Using the Socio-Technical Allocation of Resources (STAR) approach to support chronic obstructive pulmonary disease management resource allocation in integrated care systems in England
Source: Res Health Serv Reg. 2025 Dec 16;4:22. doi: 10.1007/s43999-025-00083-z (PMC12708464; doi:10.1007/s43999-025-00083-z)
Supplement: Supplementary file 1 — Supplementary Material 1 [file 43999_2025_83_MOESM1_ESM.docx]

Supplemental Table 1. Parameters used to calculate the efficiency frontier ranked by cost-effectiveness (highest first): Birmingham and Solihull ICS

| Rank | Pathway component | RBS | N treated | N who benefit | Cost per person (£) | PHB | Total cost (£) |
| --- | --- | --- | --- | --- | --- | --- | --- |
| 1 | Smoking cessation—Quit with Bella app | 100 | 715 | 139 | 13.98 | 13,900 | 9,995.70 |
| 2 | Primary care case management | 40 | 13,964 | 13,964 | 39.23 | 558,560 | 547,807.72 |
| 3 | Influenza vaccination | 75 | 6,103 | 617 | 9.58 | 46,275 | 58,466.74 |
| 4 | Secondary care outpatient appointment | 59 | 3,223 | 3,223 | 134.39 | 190,157 | 433,138.97 |
| 5 | Primary care health checks | 10 | 22,239 | 22,239 | 25.00 | 222,390 | 555,975.00 |
| 6 | Pneumonia vaccination | 75 | 1,041 | 130 | 30.00 | 9,750 | 31,230.00 |
| 7 | COVID-19 vaccination | 75 | 13,286 | 1,343 | 25.16 | 100,725 | 334,275.76 |
| 8 | Primary care case management—AECOPD | 10 | 5,850 | 5,850 | 45.19 | 58,500 | 264,361.50 |
| 9 | Spirometry testing (GP) | 75 | 1,518 | 409 | 72.00 | 30,675 | 109,296.00 |
| 10 | Spirometry testing (diagnostic hubs) | 75 | 850 | 194 | 72.00 | 14,550 | 61,200.00 |
| 11 | Spirometry testing (secondary care) | 75 | 1,262 | 340 | 122.61 | 25,500 | 154,733.82 |
| 12 | Pulmonary rehabilitation | 89 | 499 | 254 | 346.00 | 22,606 | 172,654.00 |
| 13 | GP-provided palliative care | 87 | 399 | 399 | 941.52 | 34,713 | 375,666.48 |
| 14 | Emergency attendances | 15 | 3,153 | 3,153 | 194.13 | 47,295 | 612,091.89 |
| 15 | Smoking cessation (primary prevention) | 100 | 13,649 | 3,901 | 411.50 | 390,100 | 5,616,563.50 |
| 16 | Virtual ward—early discharge | 35 | 308 | 308 | 1,199.24 | 10,780 | 369,365.92 |
| 15 | Hospital admission | 90 | 2,795 | 2,795 | 2,940.60 | 251,550 | 8,218,977.00 |
| 16 | Hospice-provided palliative care | 87 | 305 | 305 | 38,469.30 | 26,535 | 11,733,136.50 |
| 17 | Lung volume reduction surgery | 80 | 5 | 4 | 14,239.03 | 320 | 71,195.15 |

AECOPD, acute exacerbation of chronic obstructive pulmonary disease; PHB, population health benefit; RBS, relative benefit score.

Supplemental Table 2. Parameters used to calculate the efficiency frontier ranked by cost-effectiveness (highest first): Coventry Place

| Rank | Pathway component | RBS | N treated | N who benefit | Cost per person (£) | PHB | Total cost (£) |
| --- | --- | --- | --- | --- | --- | --- | --- |
| 1 | Primary care management—AECOPD | 80 | 3,115 | 3,115 | 45.19 | 249,200 | 140,766.85 |
| 2 | Primary care management | 60 | 2,036 | 2,036 | 39.23 | 122,160 | 79,872.28 |
| 3 | Influenza vaccination | 87 | 4,773 | 482 | 9.58 | 41,934 | 45,725.34 |
| 4 | COVID-19 vaccination | 87 | 4,297 | 434 | 15.00 | 37,758 | 64,455.00 |
| 5 | Pneumonia vaccination | 87 | 3,576 | 447 | 30.00 | 38,889 | 107,280.00 |
| 6 | Community COPD service | 92 | 1,711 | 1,711 | 266.00 | 157,412 | 455,126.00 |
| 7 | Spirometry (GP/Community COPD) | 98 | 2,587 | 728 | 97.85 | 71,344 | 253,138 |
| 8 | Secondary care outpatient appointment | 87 | 2,509 | 2,509 | 377.07 | 218,283 | 946,068.63 |
| 9 | Ambulatory oxygen therapy | 70 | 38 | 38 | 388.21 | 2,660 | 14,751.98 |
| 10 | Group therapy | 90 | 60 | 60 | 500.00 | 5,400 | 30,000.00 |
| 11 | Primary prevention—smoking cessation (GP/pharmacy) | 100 | 669 | 194 | 186.60 | 19,400 | 124,835.40 |
| 12 | Tertiary prevention—smoking cessation (GP/pharmacy) | 90 | 57 | 17 | 186.60 | 1,530 | 10,636.20 |
| 13 | Primary prevention—smoking cessation (HLS) | 100 | 790 | 340 | 458.87 | 34,000 | 362,507.30 |
| 14 | Tertiary prevention—smoking cessation (HLS) | 99 | 67 | 29 | 458.87 | 2,871 | 30,744.29 |
| 15 | Virtual ward | 73 | 20 | 20 | 944.79 | 1,460 | 18,895.80 |
| 16 | Pulmonary rehabilitation | 90 | 213 | 79 | 455.00 | 7,110 | 96,915.00 |
| 16 | Long-term oxygen therapy at home | 70 | 196 | 196 | 1051.00 | 13,720 | 205,996.00 |
| 17 | Post-discharge support | 73 | 311 | 69 | 266.39 | 5,037 | 82,847.29 |
| 18 | Virtual ward | 73 | 20 | 20 | 944.79 | 1,460 | 18,895.80 |
| 19 | Warm home schemes | 87 | 32 | 32 | 2763.00 | 2,784 | 88,416.00 |
| 20 | Hospital admission | 40 | 680 | 680 | 2,573.00 | 27,200 | 1,749,640.00 |

AECOPD, acute exacerbation of chronic obstructive pulmonary disease; HLS, Healthy Lifestyle service; PHB, population health benefit; RBS, relative benefit score.

Supplemental Table 3. Parameters used to calculate the efficiency frontier ranked by cost-effectiveness (highest first): Gloucestershire ICS

| Rank | Pathway component | RBS | N treated | N who benefit | Cost per person (£) | PHB | Total cost (£) |
| --- | --- | --- | --- | --- | --- | --- | --- |
| 1 | Primary care management—AECOPD | 55 | 1,134 | 1,134 | 45.19 | 62,370 | 51,245.46 |
| 2 | Pneumonia vaccination | 75 | 7,295 | 912 | 10.06 | 68,400 | 73,387.70 |
| 3 | Influenza vaccination | 75 | 11,851 | 1,197 | 10.06 | 89,775 | 119,221.06 |
| 4 | COVID-19 vaccination | 75 | 11,678 | 1,180 | 10.06 | 88,500 | 117,480.68 |
| 5 | Secondary care outpatient appointment | 50 | 2,084 | 2,084 | 133.60 | 104,200 | 278,422.40 |
| 6 | Primary care case management | 35–75 | 7,209 | 7,209 | 150.00 | 396,495c | 1,081,350.00 |
| 7 | Smoking cessation (HLS) | 100 | 1,515 | 1,078 | 217.75 | 107,800 | 329,891.25 |
| 8 | Spirometry testing (GP) | 75 | 2,005 | 147 | 17.37 | 11,025 | 34,826.85 |
| 9 | Mindsong^a^ | 90 | 80 | 80 | 284.00 | 7,200 | 22,720.00 |
| 10 | Oxygen assessment | 90 | 301 | 301 | 318.00 | 27,090 | 95,718.00 |
| 11 | Smoking cessation (GP) | 100 | 367 | 188 | 206.59 | 18,800 | 75,818.53 |
| 12 | Respiratory and Home Oxygen Assessment Service | 70 | 983 | 983 | 318.00 | 68,810 | 312,594.00 |
| 13 | KiActiv^b^ | 45 | 96 | 62 | 160.55 | 2,790 | 15,412.80 |
| 14 | Warm Home Prescriptions | 90 | 150 | 150 | 600.00 | 13,500 | 90,000.00 |
| 15 | Pulmonary rehabilitation | 90 | 600 | 400 | 954.00 | 36,000 | 572,400.00 |
| 16 | Virtual ward | 62 | 827 | 827 | 1,307.38 | 51,274 | 1,081,203.26 |
| 17 | Spirometry testing—secondary care | 75 | 423 | 31 | 174.39 | 2,325 | 73,766.97 |
| 18 | Hospital admission | 59 | 652 | 652 | 2,490.88 | 38,468 | 1,624,053.76 |
| 19 | Lung volume reduction surgery | 20–40 | 5 | 4 | 9,804.33 | 120^c^ | 49,021.65 |

^a^Mindsong is a ‘breath in, sing out course’ for people with chronic lung conditions.
^b^KiActiv is a personalised online physical activity course for patients who are referred by primary, community and secondary care.
^c^Based on the midpoint of the RBS range
AECOPD, acute exacerbation of chronic obstructive pulmonary disease; HLS, Healthy Lifestyle service; PHB, population health benefit; RBS, relative benefit score.

Supplemental Table 4. Parameters used to calculate the efficiency frontier ranked by cost-effectiveness (highest first): Northamptonshire ICS

| Rank | Pathway component | RBS | N treated | N who benefit | Cost per person (£) | PHB | Total cost (£) |
| --- | --- | --- | --- | --- | --- | --- | --- |
| 1 | NSport activity on referral^a^ | 89 | 84 | 64 | 40.38 | 5.696 | 3,391.92 |
| 2 | Primary care management of AECOPD | 67 | 5,234 | 5,234 | 45.19 | 350,678 | 236,524.46 |
| 3 | Pneumonia vaccination | 87 | 1,947 | 243 | 10.00 | 21,141 | 19,470.00 |
| 4 | Group therapy (Breathing Space) | 95 | 2,112 | 2,112 | 99.43 | 200,640 | 209,996.16 |
| 5 | Influenza vaccination | 87 | 6,926 | 700 | 9.58 | 60,900 | 66,351.08 |
| 6 | Primary care case management | 50 | 6,626 | 6.626 | 61.85 | 331,300 | 409,818.10 |
| 7 | COVID-19 vaccination | 87 | 10,630 | 1,074 | 15.00 | 93,438 | 159,450.00 |
| 8 | Primary prevention—smoking cessation | 100 | 2,728 | 1,676 | 215.00 | 167,600 | 586,520.00 |
| 9 | Secondary care outpatient appointment | 85 | 4,690 | 4,200 | 335.25 | 357,000 | 1,572,322.50 |
| 10 | Tertiary prevention—smoking cessation | 75 | 372 | 229 | 215.00 | 17,175 | 79,980.00 |
| 11 | Emergency attendees | 40 | 2,205 | 2,205 | 198.30 | 88,200 | 437,251.50 |
| 12 | Spirometry (secondary care) | 98 | 4,608 | 559 | 72.00 | 54,782 | 331,776.00 |
| 13 | Spirometry (GP) | 98 | 8,591 | 1,042 | 72.00 | 102,116 | 618,552.00 |
| 14 | Pulmonary rehabilitation (ROCKET/RESTART)^b^ | 92 | 1,287 | 202 | 148.50 | 18,584 | 191,119.50 |
| 15 | Northampton Energy Saving Service | 25 | 32 | 32 | 618.58 | 800 | 19,794.56 |
| 16 | Community COPD service—ROCKET/RESTART (hospital avoidance)^b^ | 80 | 1,546 | 344 | 429.00 | 27,520 | 663,234.00 |
| 17 | Hospital admission | 50 | 725 | 725 | 2,304.66 | 36,250 | 1,670,878.50 |
| 18 | Lung volume reduction surgery | 28 | 10 | 8 | 14,285.22 | 224 | 142,852.20 |
| 19 | Lung transplant | 37 | 1 | 1 | 89,477.00 | 37 | 89,477.00 |

^a^NSport (Northamptonshire Sport) is a physical activity, health and wellbeing charity.
^b^ROCKET and RESTART are community COPD services based at Northampton and Kettering General Hospitals.
AECOPD, acute exacerbation of chronic obstructive pulmonary disease; PHB, population health benefit; RBS, relative benefit score.

Supplemental Table 5. Parameters used to calculate the efficiency frontier ranked by cost-effectiveness (highest first): Nottingham and Nottinghamshire ICS

| Rank | Pathway component | RBS | N treated | N who benefit | Cost per person (£) | PHB | Total cost (£) |
| --- | --- | --- | --- | --- | --- | --- | --- |
| 1 | Group therapy—Breathe Easy | 62 | 175 | 175 | 31.03 | 10,850 | 5,430.25 |
| 2 | Primary care health checks | 65 | 11,410 | 11,410 | 33.00 | 741,650 | 376,530.00 |
| 3 | Primary care management—case management | 75 | 12,148 | 12,148 | 39.23 | 911,100 | 476,566.04 |
| 4 | Primary care management—AECOPD | 70 | 5,511 | 5,511 | 45.19 | 385,770 | 249,042.09 |
| 5 | IAPT services | 90 | 215 | 215 | 86.00 | 19,350 | 18,490.00 |
| 6 | Influenza vaccination | 90 | 20,515 | 2,072 | 9.58 | 186,480 | 196,533.70 |
| 7 | Oxygen therapy (long-term) | 90 | 378 | 378 | 96.00 | 34,020 | 36,288.00 |
| 8 | Primary prevention—smoking cessation | 100 | 4,824 | 2,833 | 74.52 | 283,300 | 359,484.48 |
| 9 | Tertiary prevention—smoking cessation | 98 | 1,865 | 1,088 | 74.52 | 106,624 | 138,979.80 |
| 10 | COVID-19 vaccination | 90 | 22,105 | 2,234 | 15.00 | 201,060 | 331,575.00 |
| 11 | INTENT smoking cessation in schools^a^ | 90 | 13,637 | 419 | 6.30 | 37,710 | 85,913.10 |
| 12 | Pneumonia vaccination | 90 | 14,165 | 1,771 | 30.00 | 159,390 | 424,950.00 |
| 13 | Spirometry (GP) | 95 | 1,955 | 541 | 72.00 | 51,395 | 143,640.00 |
| 14 | Spirometry (secondary care) | 95 | 3,770 | 1,044 | 89.40 | 99,180 | 337,038.00 |
| 15 | Secondary care outpatient appointment/Respiratory physiology | 33 | 12,865 | 12,865 | 119.00 | 424,545 | 1,530,935.00 |
| 16 | Oxygen therapy (ambulatory) | 90 | 436 | 436 | 623.00 | 39,240 | 271,628.00 |
| 17 | Emergency attendances | 23 | 1,735 | 1,735 | 205.20 | 39,905 | 356,022.00 |
| 18 | Pulmonary rehabilitation | 90 | 2,249 | 901 | 346.00 | 81,090 | 778,154.00 |
| 19 | Community COPD services | 95 | 2,427 | 2,427 | 1,477.04 | 230,565 | 3,584,776.08 |
| 20 | Warm homes | 65 | 39 | 39 | 2,370.97 | 2,535 | 92,467.83 |
| 21 | Respiratory assessment unit | 30 | 1,152 | 1,152 | 3,668.86 | 34,560 | 4,226,526.72 |
| 22 | Hospital admission | 23 | 2,135 | 2,135 | 2,856.00 | 49,105 | 6,097,560.00 |
| 23 | Lung volume reduction surgery | 13 | 5 | 4 | 7,700.00 | 52 | 38,500.00 |

^a^INTENT is a smoking prevention programme.
AECOPD, acute exacerbation of chronic obstructive pulmonary disease; IAPT, improving access to psychological therapies; PHB, population health benefit; RBS, relative benefit score.

**Supplemental Figure 1.** The current care pathways for those living with and at risk of developing COPD in each ICS

**A.** Birmingham and Solihull ICS


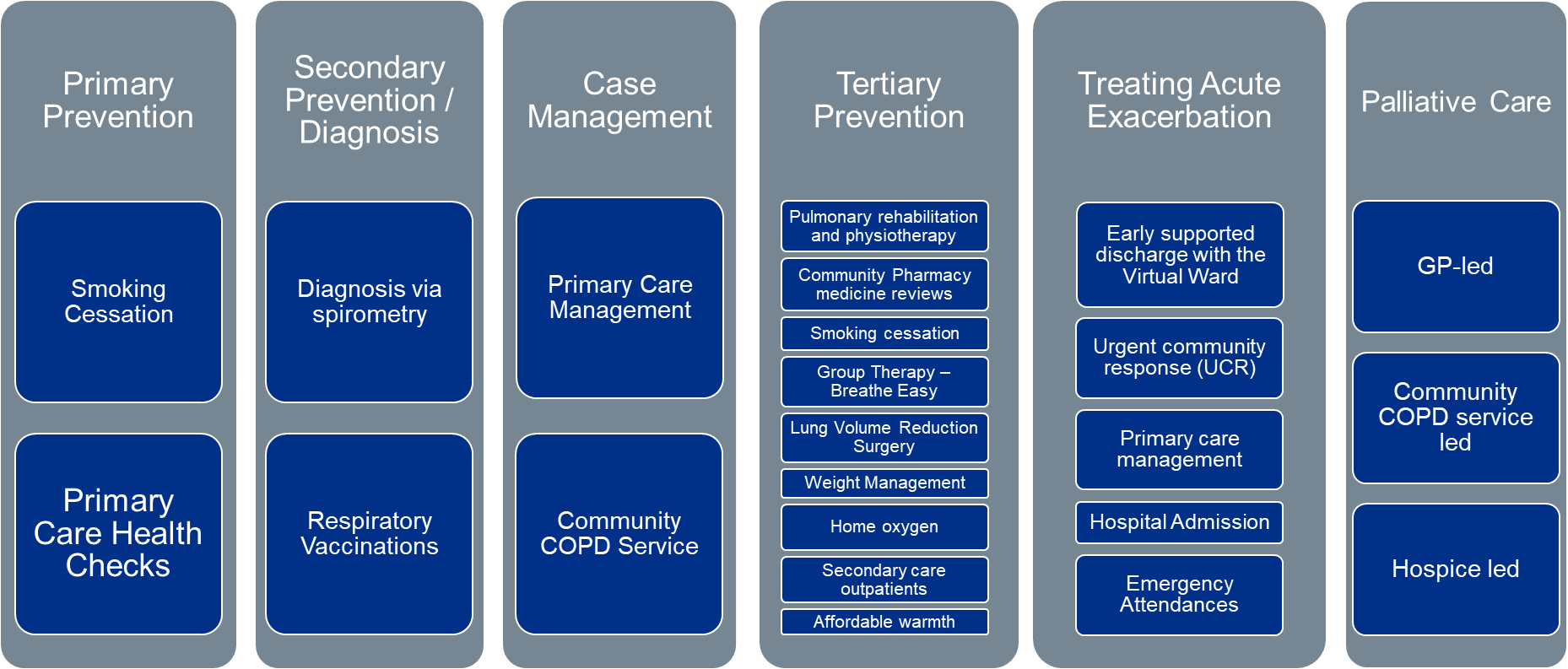


**B.** Coventry Place


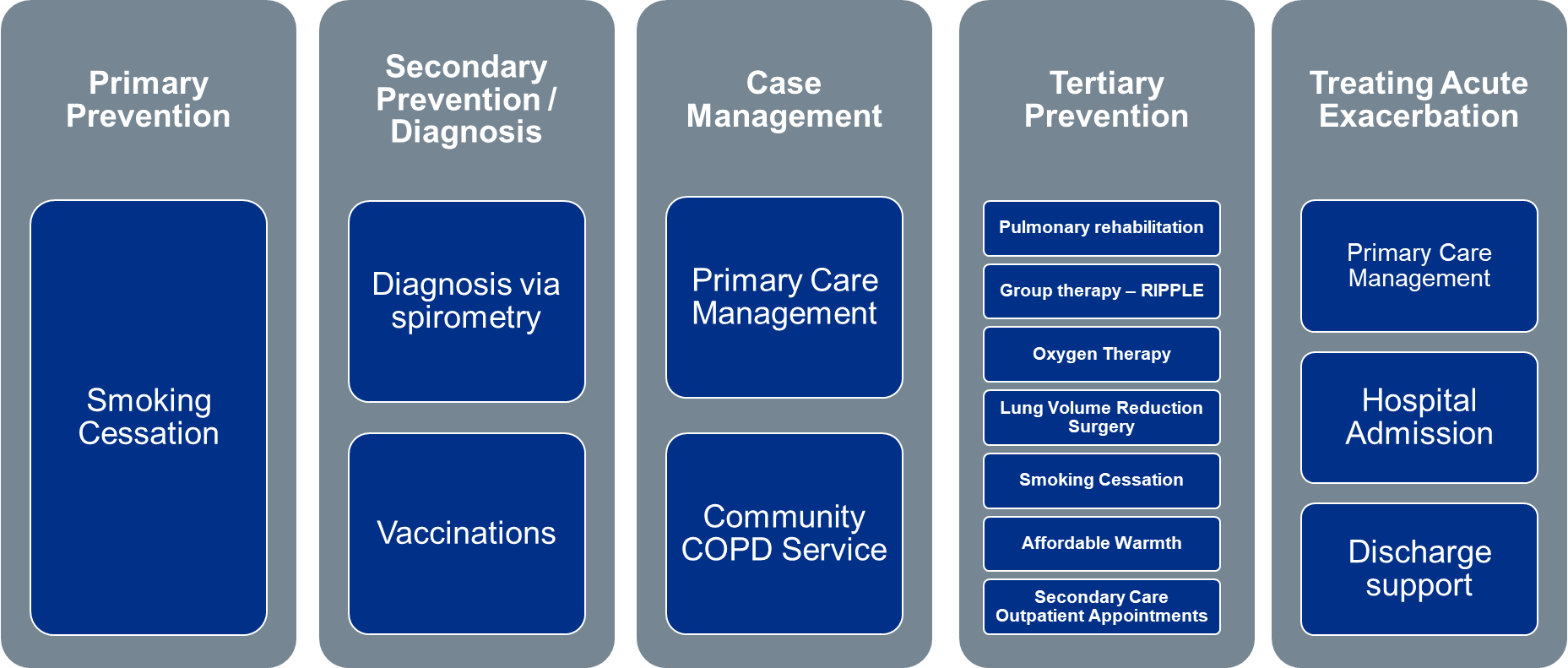


**C.** Gloucestershire ICS


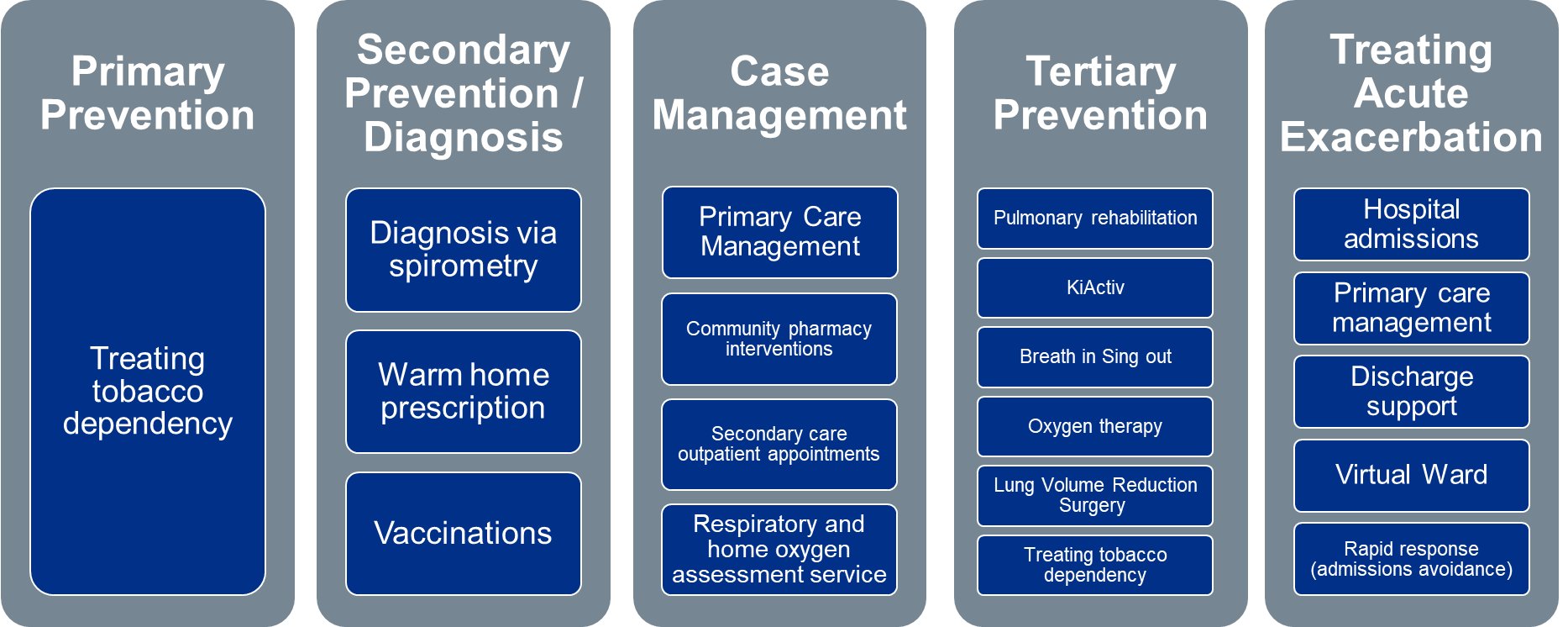


**D.** Northamptonshire ICS


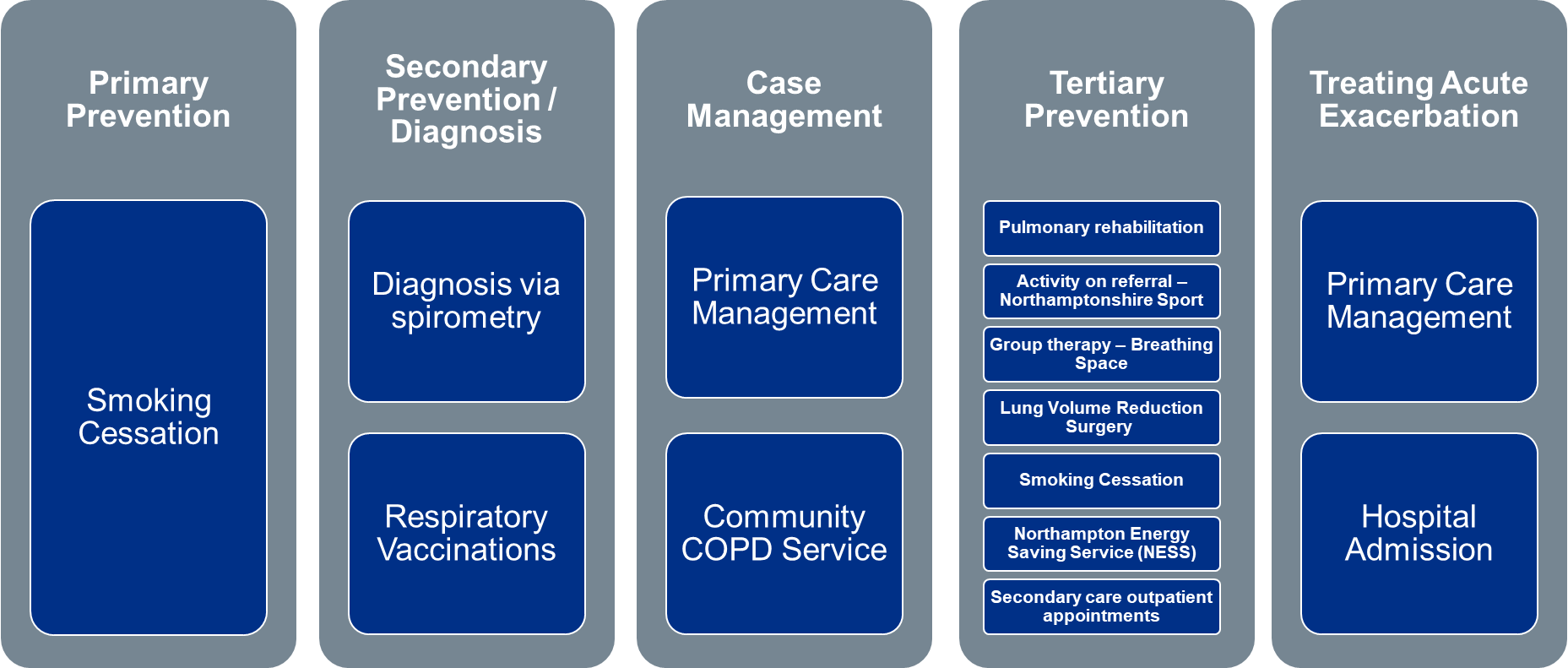


**E.** Nottingham and Nottinghamshire ICS


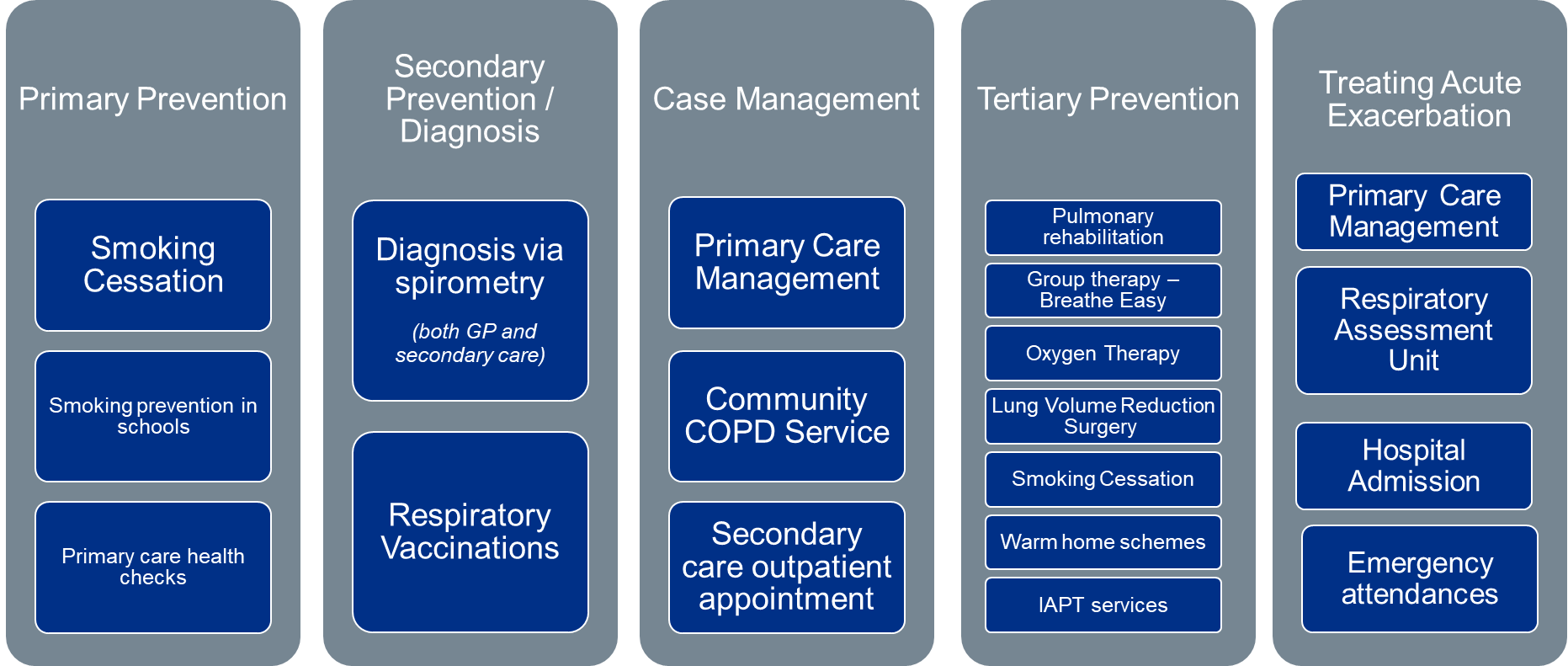


**Supplemental Figure 2.** COPD population pyramids in each ICS

Estimates for the size of the COPD population in each ICS were based on government health statistics and published literature.

**A.** Birmingham and Solihull ICS


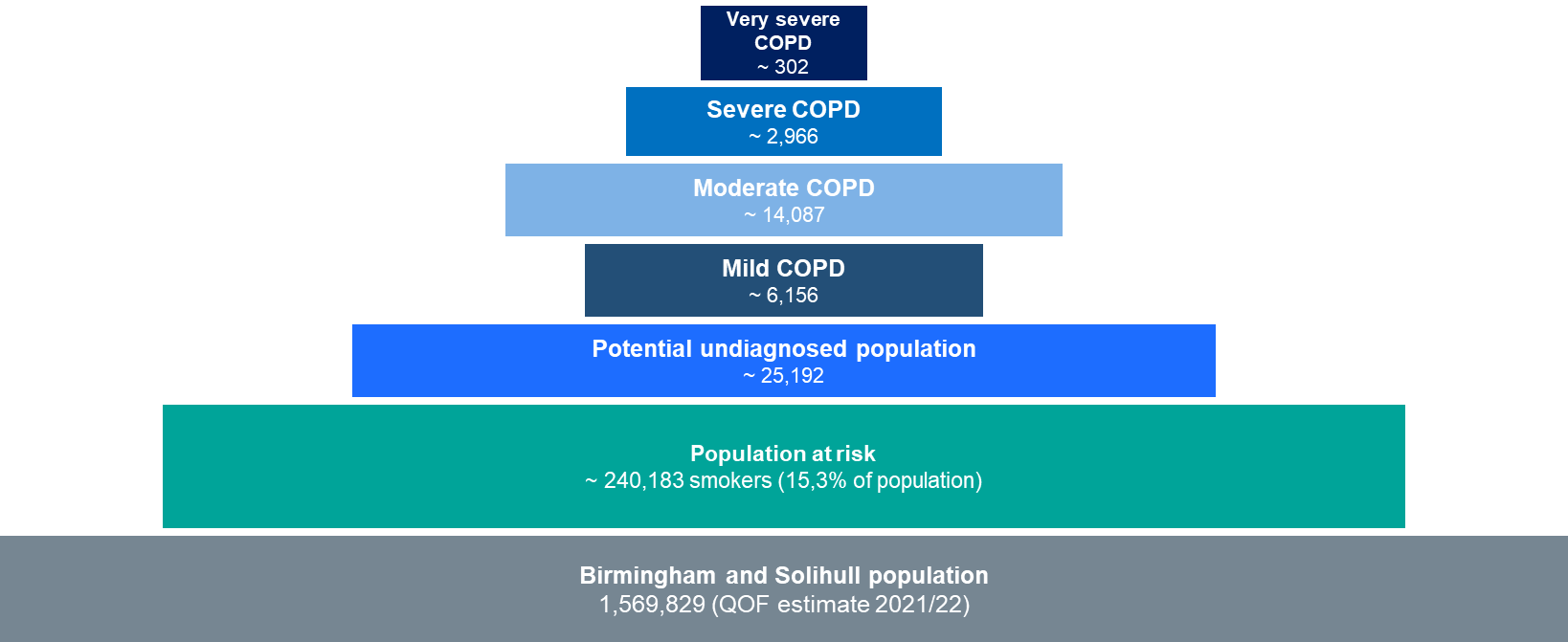


**B.** Coventry Place


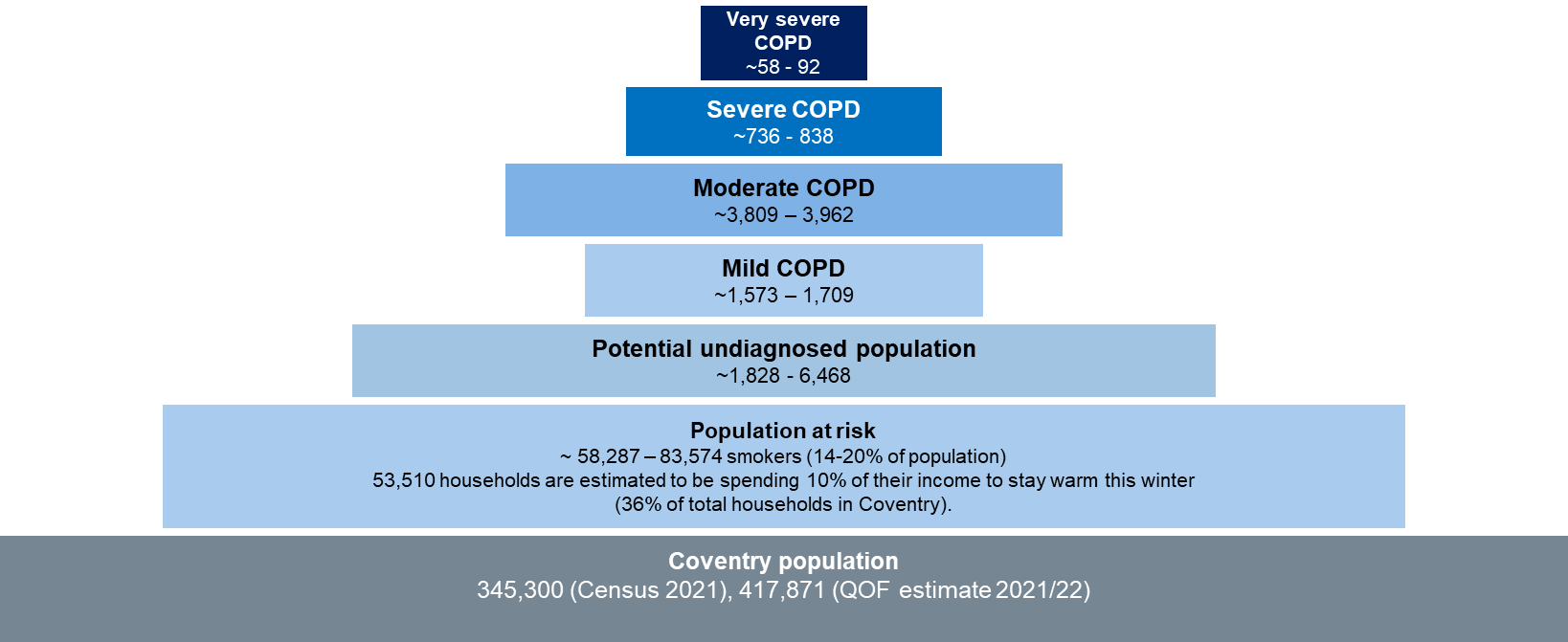


**C.** Gloucestershire ICS

***
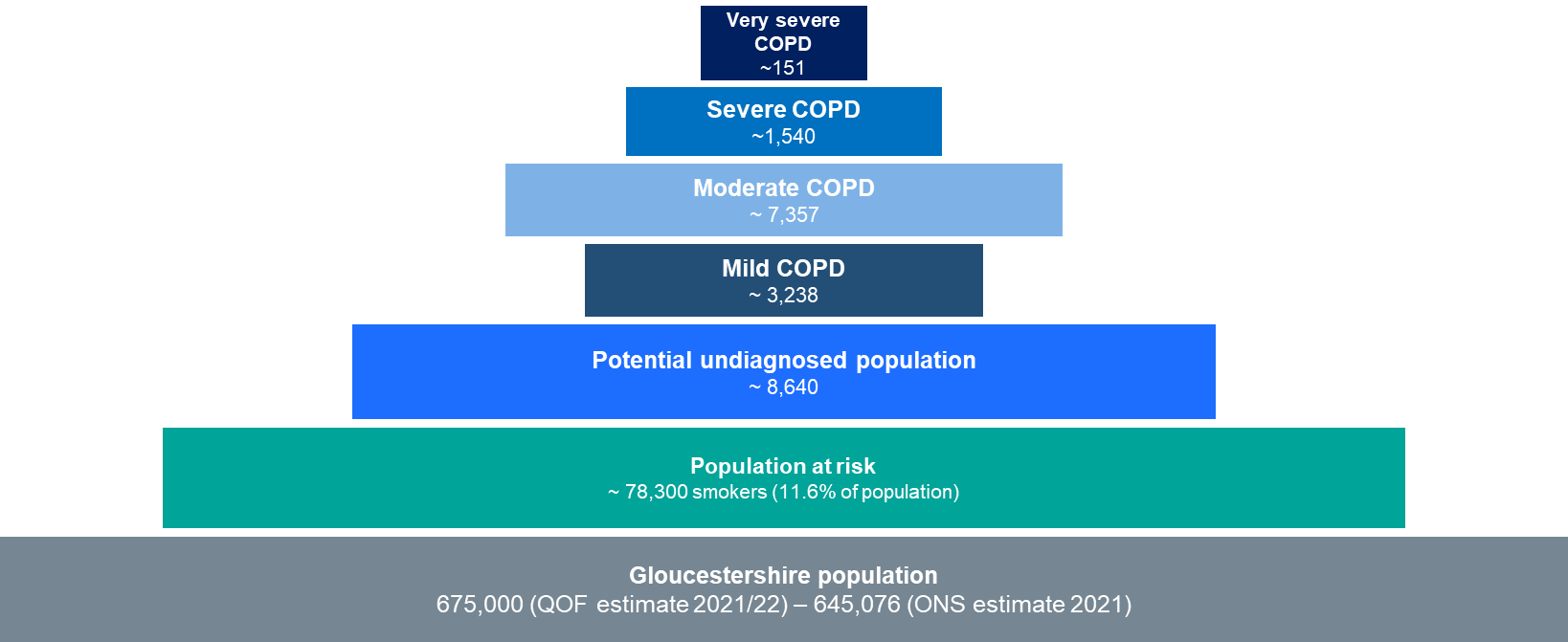
***

**D.** Northamptonshire ICS


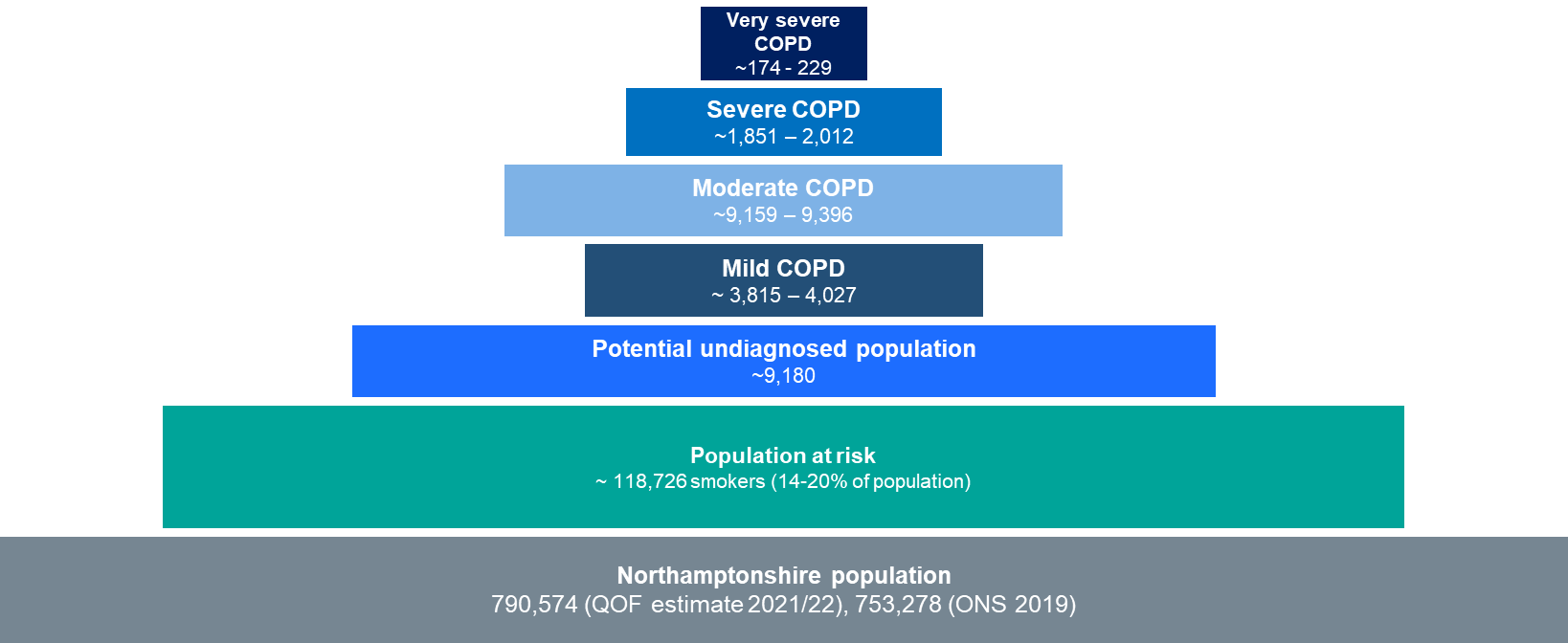


**E.** Nottingham and Nottinghamshire ICS


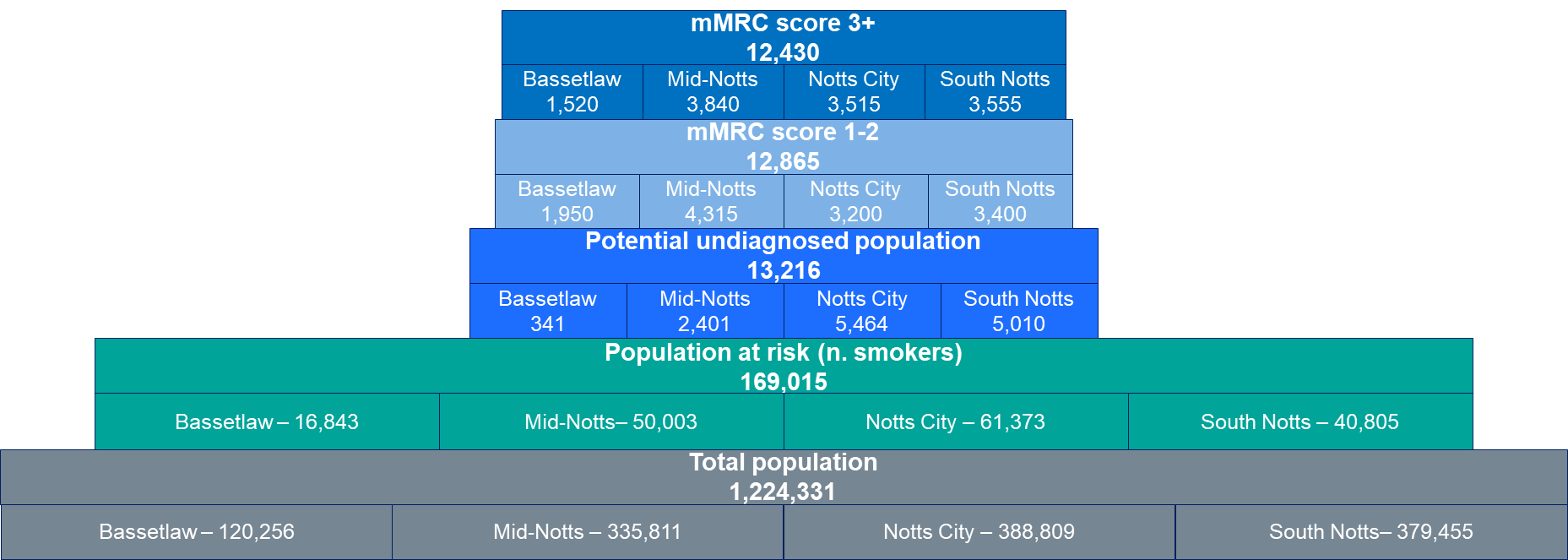


**Supplemental Figure 3.** Benefit scores of each of the interventions in the current COPD pathway generated in the decision conferences.

**A.** Birmingham and Solihull ICS


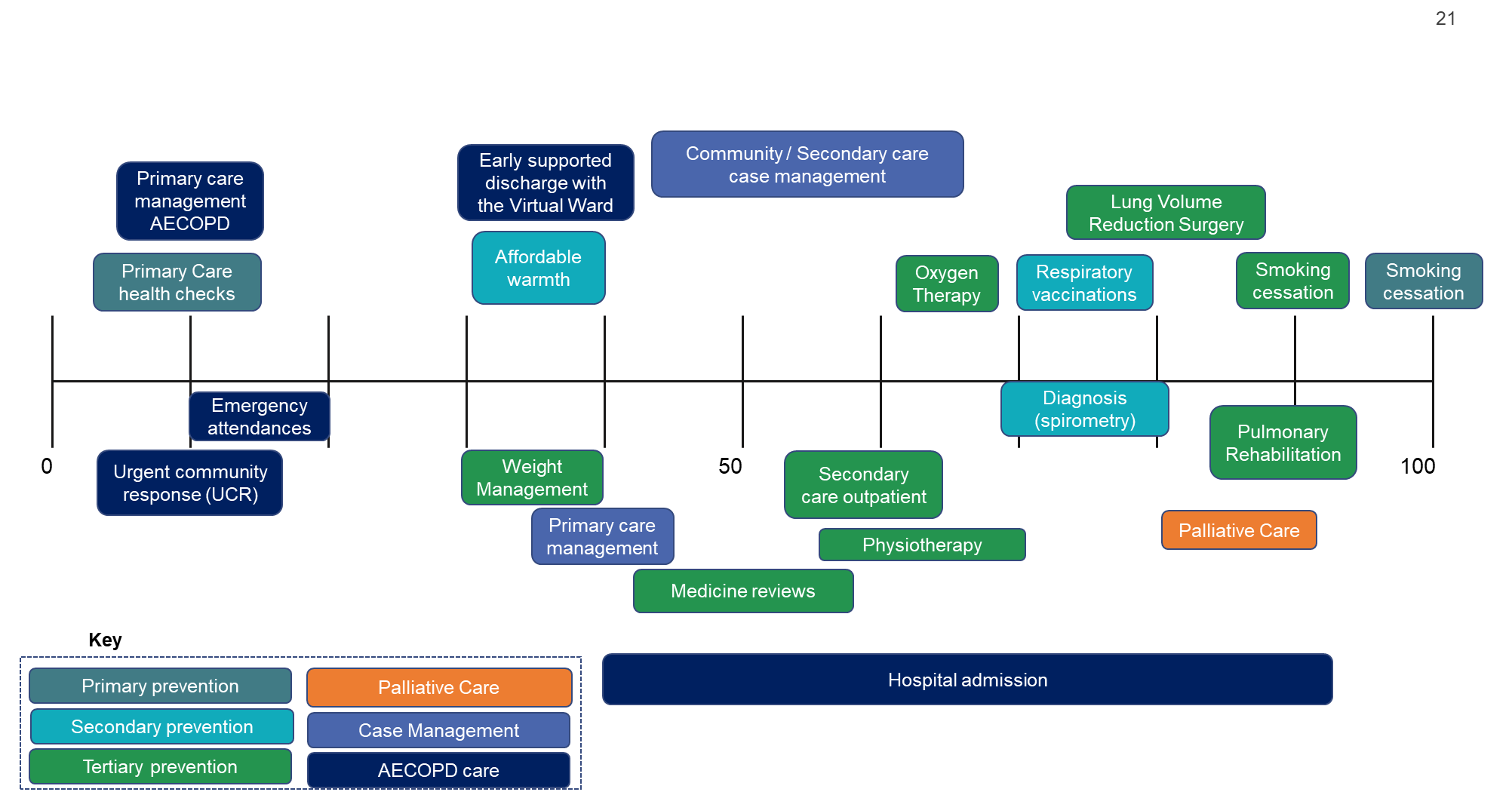


**B.** Coventry Place


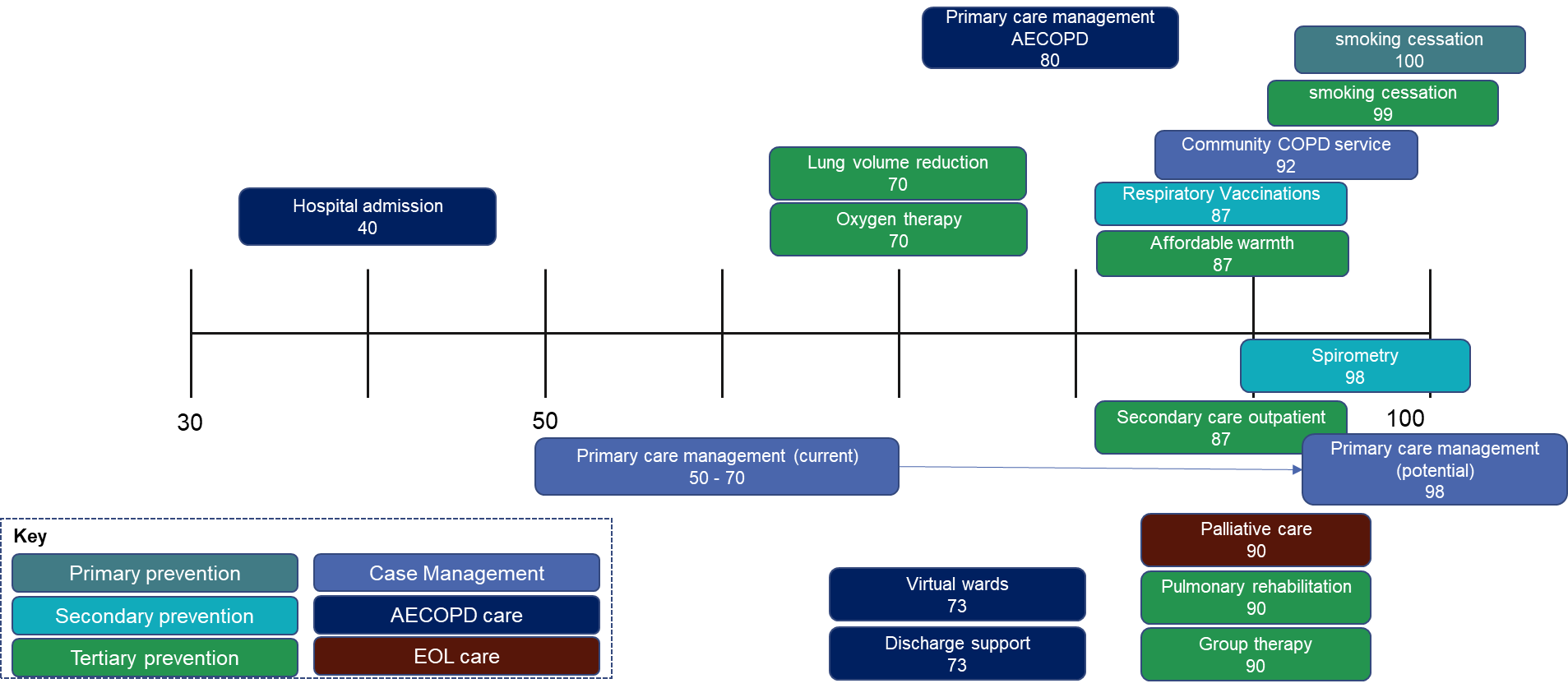


**C.** Gloucestershire ICS


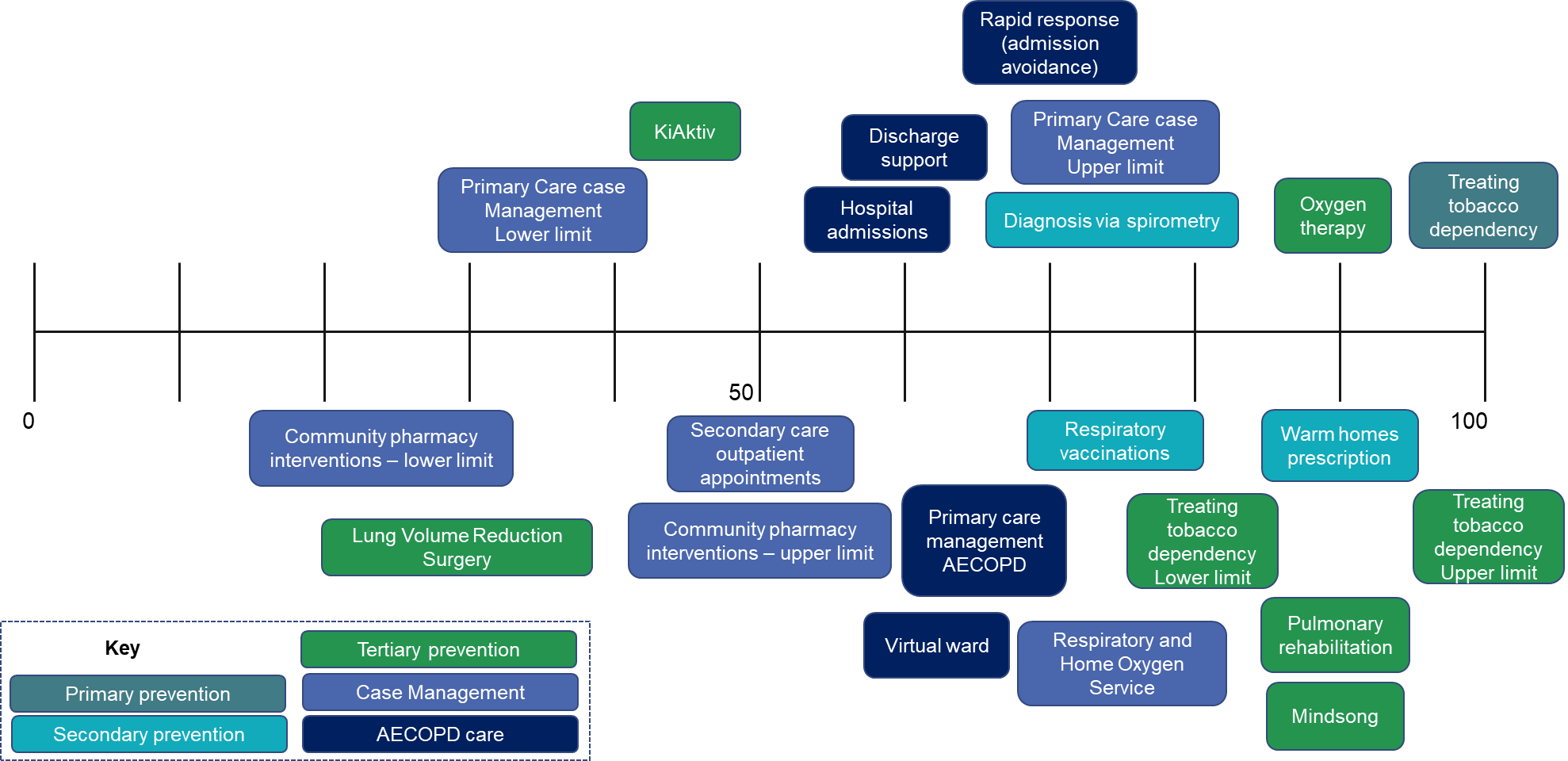


**D.** Northamptonshire ICS


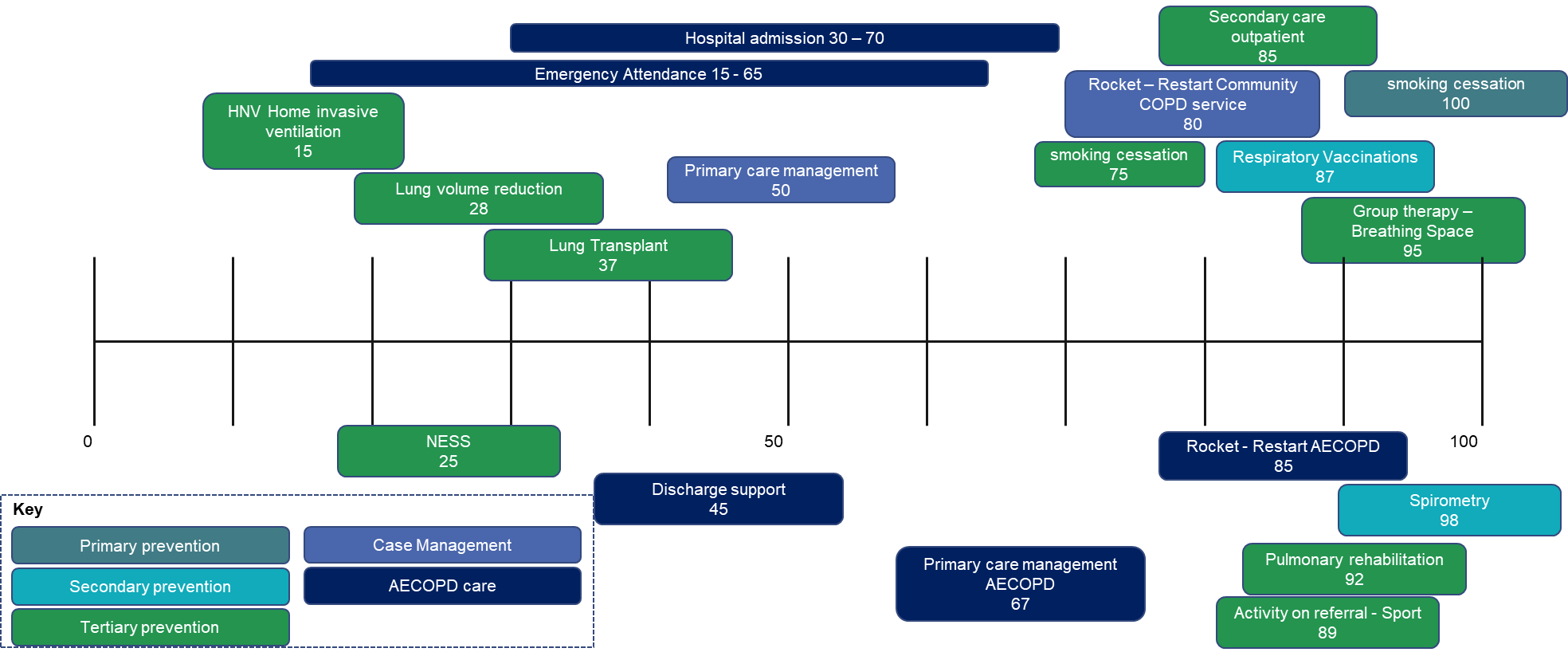


**E.** Nottingham and Nottinghamshire ICS


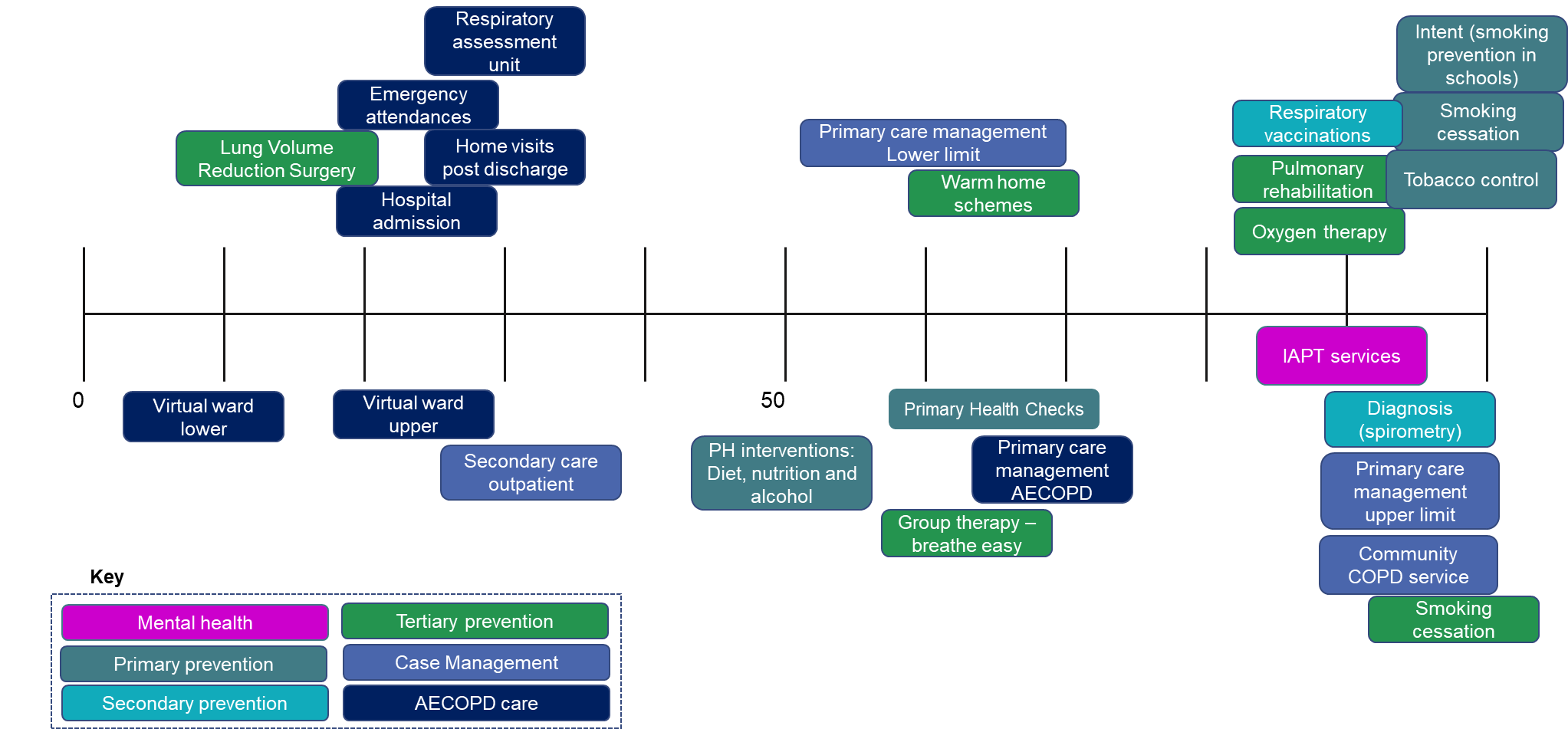


**Supplemental Figure 4.** Efficiency frontiers for the current COPD pathways

**A.** Birmingham and Solihull ICS


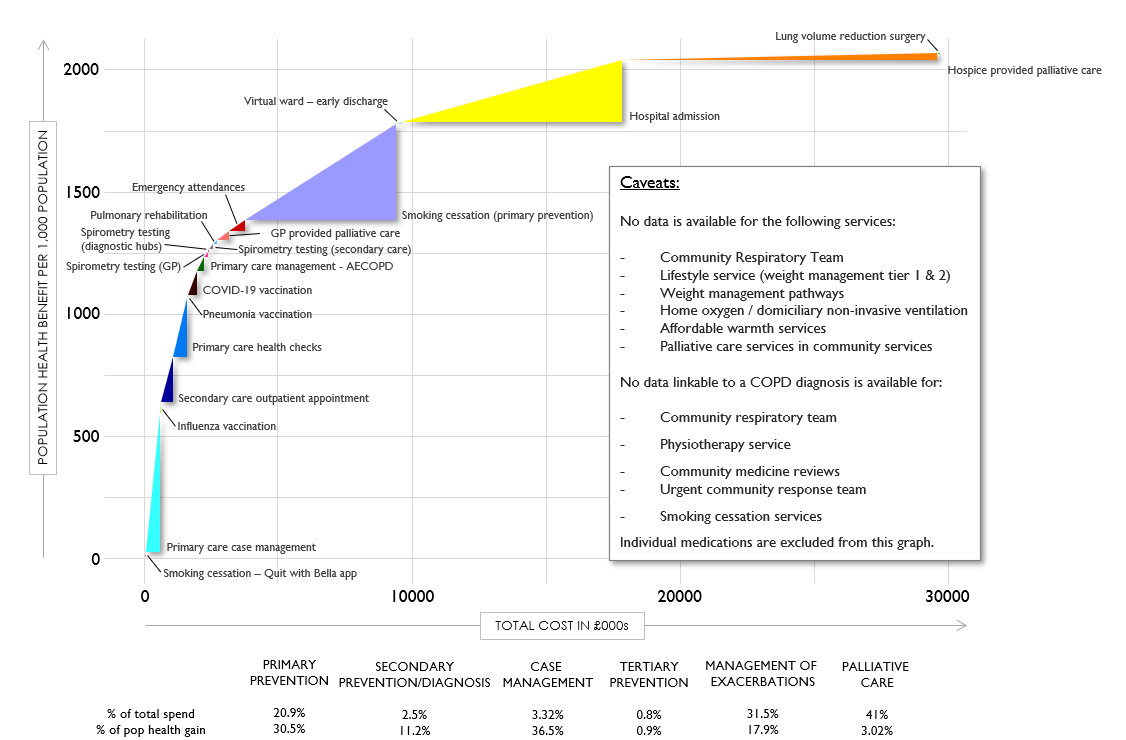


**B.** Coventry Place


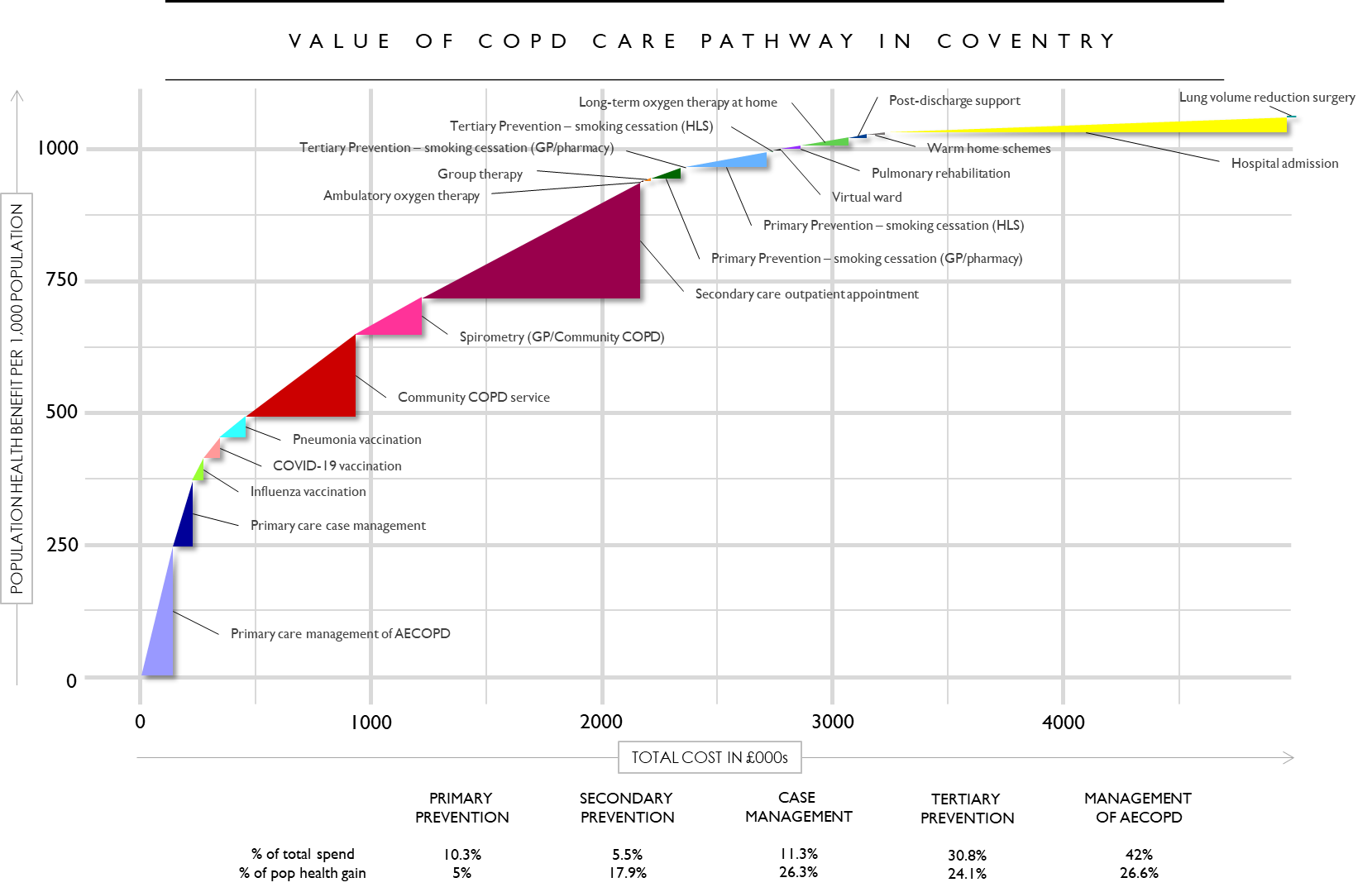


**C.** Gloucestershire ICS


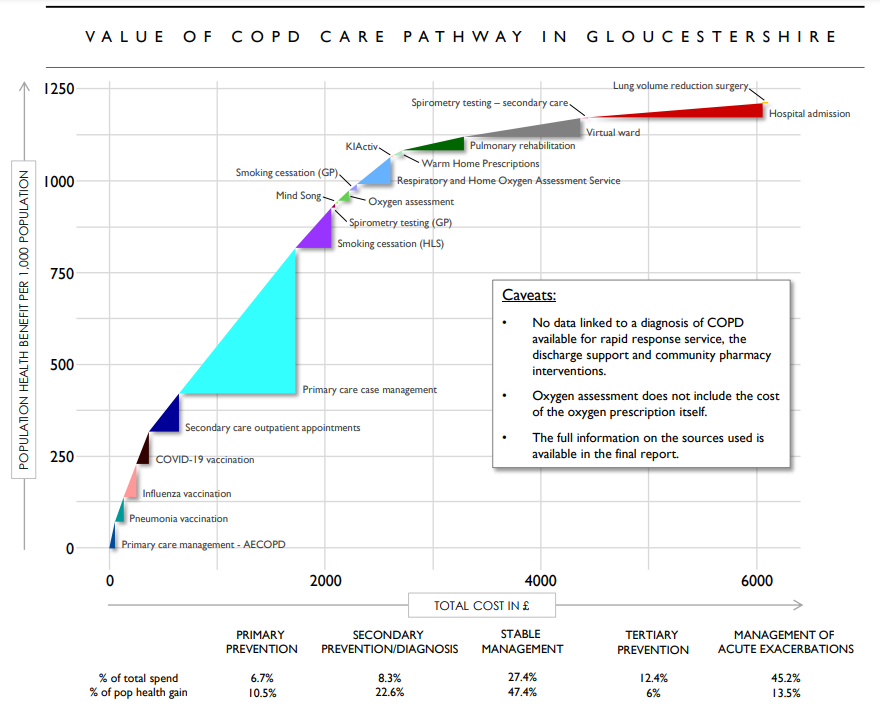


**D.** Northamptonshire ICS


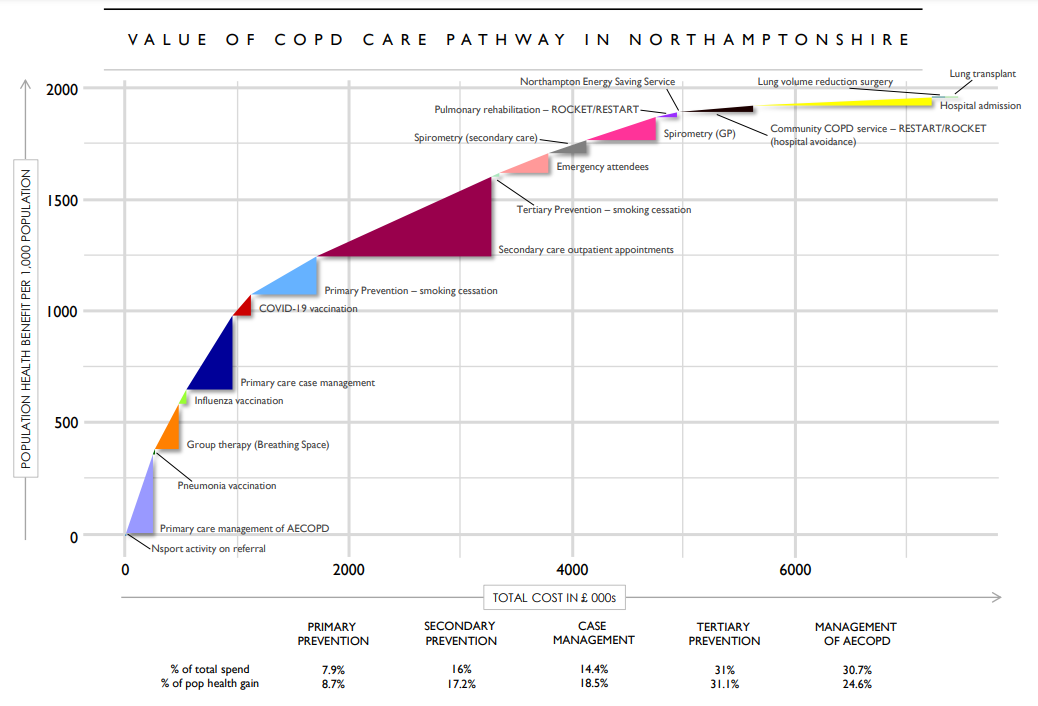


**SUPPLEMENTAL APPENDIX**

**Valuing the current COPD pathway**

A visual representation of the costs and population health benefits of the current COPD pathway (an “efficiency frontier”) in each ICS was created following the first decision conference.

Population health benefit (*N_j_***B_j_*) was the product of the number (*N_j_*) of patients who benefited from an intervention (indexed by *j)* in 2021/22 and the relative benefit (*B_j_*) taken from the decision conferences. As the purpose of this exercise was to make a decisions about interventions in the current care pathway, the relative benefit is a sufficient proxy for the absolute benefit for the purpose of this exercise. The number who benefit (*N_j_*) was calculated using the number of people treated and the numbers needed to treat. The number of people treated was collected from routinely collected data from the ICS and validated in the decision conferences. The number needed to treat was informed by estimates from the umbrella literature review or NICE guidance^26^ .

Costs (*C_j_*) were the estimated annual cost of the intervention in 2021/22. Ahead of the workshops, each ICS was asked to provide actual costs of treatment or service provision, including 1) costs that would be incurred by the relevant commissioners, whether that be the ICS, the local authority or others, and 2) for voluntary service provision, the cost to the organisation providing the service.

**Interpreting the efficiency frontier**

The efficiency frontier is made up of triangles representing the value for money of each intervention in that pathway (Figure 1). This allows us to visually compare the impact of different interventions and programmes across the whole pathway (e.g., spirometry and pulmonary rehabilitation). The y-axis shows the expected population health benefit for an intervention (the product of the number who benefit and the benefit score) compared with current care. The x-axis displays the estimated annual cost for an intervention.

***Figure 1. Populating the efficiency frontier***

STAR’s visual models are what makes it applied common sense. In Figure 2 below, we can see at a glance that the triangle on the right represents an intervention that is much more cost-effective than the intervention represented by the triangle on the left: as we increase spending, the benefits increase quickly for the triangle on the right but only slowly for the triangle on the left.

***Figure 2. Triangles showing low value for money (left) and high value for money (right).***

The triangles are then ordered according to their value for money to display the ‘efficiency frontier’. This shows either where there are opportunities to spend the existing money in a different way to provide more value for money, or where additional investment will be best targeted. The purpose of the efficiency frontier is to help stakeholders think about how the care pathway for COPD ought to be developed. The aim is to move the curve to the left and upwards (represented in Figure 3a), thus reducing costs and improving the population health benefit of the pathway (compared with the curve in Figure 3b).

*Figure 3. Different efficiency frontiers with good (a) and bad (b) value for money.*

(a) (b)


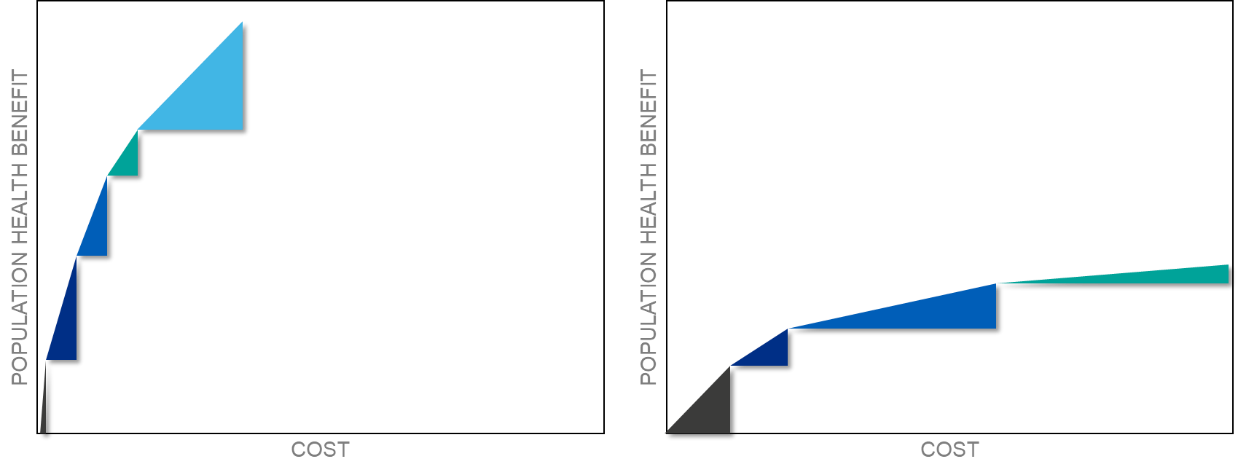


Different efficiency frontiers with good (a) and bad (b) value for money.

**Allocation model calculations**

Additional population health benefit due to pathway improvement (PHB) was represented as: 𝑃𝐻𝐵_𝑗+𝑘+𝑖_= 𝑁_𝑗_×𝐵_𝑗_+𝑁_𝑖_×𝐵_𝑖_+𝑁_𝑘_×𝐵_𝑘_…, where *j*, *i* and *k* represent each intervention in the pathway improvement. *N_j_* is the number of individuals who would benefit from the intervention *j* each year and *B_j_* is the potential benefit in quality and length of life, assuming successful implementation, to the typical beneficiary, compared with current care. The benefit from intervention *j* consists of direct health benefit in terms of length and quality of life from the intervention itself, as defined by participants in the decision conferences.

In some cases, the pathway improvement may have led to a decrease in activity in another pathway component. In this case, the lost population health benefit that would have been generated by this pathway component was included in the calculation: 𝑃𝐻𝐵 = 𝑁_𝑗_×𝐵_𝑗_−𝑁_𝑙𝑖_×𝐵_𝑖_… where *N_l_* is the number of people who would now not be treated due to implementation of the improvement.

The additional costs of pathway improvement (*N_t_C*) were calculated as: 𝑁_𝑡_𝐶= 𝑁_𝑡𝑗_×𝐶_𝑗_+𝑁_𝑡𝑖_×𝐶_𝑖_+𝑁_𝑡𝑘_×𝐶_𝑘_..., where *N_tj_* is equal to the number of individuals who are expected to be treated by the intervention *j* within a given year and *C_j_* is equal to the expected average cost of the intervention for treating one individual. It was assumed that costs applied to each person treated and that there is a linear relationship between costs and numbers treated.

The expected impact on healthcare resource use (*R*) elsewhere in the COPD pathway (defined as “pathway components”, including hospital admissions, GP appointments or acute exacerbations) for each pathway improvement was determined using numbers needed to treat (NNT) calculated from risk data for each intervention sourced from the umbrella literature review using the calculation $\frac{1}{absolute risk reduction}$. When information was not available in the literature, it was assumed that the improvement would not have an impact on other pathway elements. We modelled the latest timeframe in which the interventions were expected to have statistically significant effects on the rest of the pathway. The number who benefit (*N_j,i,k_…)* from each intervention in the pathway improvement was divided by the relevant NNT: 𝑅_𝑦_=𝑁_𝑗_𝑁𝑁𝑇_𝑦_, where *y* is equal to the pathway component affected by the improvement (usually hospital admissions). Due to the different timescales that primary prevention would have on the COPD pathway (through reducing the number of people developing COPD) compared with other interventions, its effects on the rest of the pathway were not included in the visualisations.

The cost savings expected for each pathway improvement were calculated by multiplying the expected impact on healthcare resource use (e.g., number of hospital admissions avoided) by the estimated costs of each component (based on tariff prices): 𝑅𝐶_𝑦+𝑥+𝑧_=𝑅_𝑦_ ×𝐶_𝑣𝑦_+ 𝑅_𝑥_× 𝐶_𝑣𝑥_+ 𝑅_𝑧_× 𝐶_𝑣𝑧_…, where *y*, *x* and *z* represent the components impacted by the improvement, and C*_v_* represents the cost of the pathway component in question.

Total additional pathway cost was equal to the additional cost of the pathway improvement to the NHS minus the cost savings: 𝑁_𝑡_𝐶−𝑅𝐶_𝑣_. Negative numbers represent cost savings and positive numbers represent additional costs. For pathway improvements that would reduce the number of people expected to get COPD in the future (i.e., primary prevention), the cost saved was estimated by multiplying the expected number of avoided cases of COPD (based on NNTs) by the expected cost of treating one person with COPD for a year.

The additional cost / additional population health ratio was calculated as: (𝑁_𝑡_𝐶−𝑅𝐶_𝑣_)/𝑃𝐻𝐵. The lower the ratio, the better, with a negative ratio representing improvements which are both cost-saving and health-generating. A ratio of 1 would mean that one additional unit of population health benefit is generated for each unit of population health benefit.

The cost ratio was calculated by dividing the cost saving by the additional cost of the pathway improvement: 𝑅𝐶_𝑣_/𝑁_𝑡_𝐶. A ratio of 1 means that the improvement is cost-neutral (i.e., £1 saved for every £1 spent elsewhere in the pathway). A ratio of 1.1 means £1.10 is saved elsewhere in the pathway for every £1 spent on the improvement. Numbers below 1 represent interventions that are cost-incurring.
